# Technological and functional analysis of 80–60 ka bone wedges from Sibudu (KwaZulu-Natal, South Africa)

**DOI:** 10.1038/s41598-022-20680-z

**Published:** 2022-09-29

**Authors:** Francesco d’Errico, Lucinda R. Backwell, Lyn Wadley, Lila Geis, Alain Queffelec, William E. Banks, Luc Doyon

**Affiliations:** 1 Université de Bordeaux, CNRS, MCC, PACEA, UMR5199, Bâtiment B2, Allée Geoffroy Saint Hilaire, CS 50023, 33600 Pessac, France; 2 Centre for Early Sapiens Behaviour (SapienCE), Department of Archaeology, History, Cultural Studies and Religion, University of Bergen, Bergen, Norway; 3 Instituto Superior de Estudios Sociales (ISES-CONICET), Córdoba 191, San Miguel de Tucumán (CP4000), Tucumán, Argentina; 4 Centre of Exploration for the Deep Human Journey, University of the Witwatersrand, Private Bag 3, Wits, Johannesburg, 2050 South Africa; 5 Evolutionary Studies Institute, University of the Witwatersrand, Private Bag 3, Wits, Johannesburg, 2050 South Africa; 6 Biodiversity Institute, University of Kansas, 1345 Jayhawk Boulevard, Lawrence, KS 66045 USA; 7 Institute of Cultural Heritage, Shandong University, Jimo-Binhai Highway 72, Qingdao, 266237 China

**Keywords:** Archaeology, Cultural evolution, Anthropology, Archaeology

## Abstract

Fully shaped, morphologically standardized bone tools are generally considered reliable indicators of the emergence of modern behavior. We report the discovery of 23 double-beveled bone tools from ~ 80,000–60,000-year-old archaeological layers at Sibudu Cave in KwaZulu-Natal, South Africa. We analyzed the texture of use-wear on the archaeological bone tools, and on bone tool replicas experimentally used in debarking trees, processing rabbit pelts with and without an ochre compound, digging in sediment in and outside a cave, and on ethnographic artefacts. Debarking trees and digging in humus-rich soil produce use-wear patterns closely matching those observed on most Sibudu tools. This tool type is associated with three different Middle Stone Age cultural traditions at Sibudu that span 20,000 years, yet they are absent at contemporaneous sites. Our results support a scenario in which some southern African early modern human groups developed and locally maintained specific, highly standardized cultural traits while sharing others at a sub-continental scale. We demonstrate that technological and texture analyses are effective means by which to infer past behaviors and assess the significance of prehistoric cultural innovations.

## Introduction

Members of our lineage have used bone to interact with their environments since at least 2 million years ago (Myr)^1–6^. These first bone tools consisted of unmodified or minimally shaped weathered bone fragments and horn cores used as digging implements. It is only by 1.8 Myr that large bone fragments were modified by knapping, the technique used to produce flaked stone tools^7,8^, and by 1.4 Myr that knapping was used to shape bone bifaces similar to their contemporary Acheulean stone counterparts^9–11^. Unmodified or marginally modified bone tools remained part of the technological repertoire of prehistoric hominins in Africa and Eurasia until relatively recently e.g.,^12–20^. It was suggested that despite their low degree of modification, some of these tools were used for specific functions^14,21,22^. A relevant, related question is when bone tools emerged that were entirely worked with techniques adapted to shaping osseous material, such as grinding, scraping, grooving and gouging. The application of these techniques to bone, antler and ivory allows the maker to determine the tool’s final shape and size with a high degree of accuracy. Sophisticated bone technology leading to the production of fully shaped bone artefacts, often called formal bone tools^23,24^, was considered until the beginning of this century an innovation of anatomically modern humans colonizing the European territories some 40 thousand years ago (ka)^25–27^. Research conducted during the last two decades has revealed instances of formal bone tools in Northwest, Central and southern Africa at sites dated from MIS5 (~ 120 ka) to MIS2 (~ 20 ka) in the Middle Stone Age (MSA) and Early Later Stone Age (ELSA).

In Northwest Africa, objects considered as the first formal bone tools consist of modified rib fragments interpreted as smoothers, i.e., tools used to process hides. They were found in the Aterian MSA at El Mnasra, layers 5 and 6 dated *c*. 107 ka^28^, and Contrebandiers Cave, sector IV layer 2 dated *c.* 95 ka^22^. At Contrebandiers Cave, modified fragments, interpreted as formal and expedient bone tools, come from layers as old as 120 ka^22^. Other fragments of ribs split longitudinally and partially thinned by scraping and grinding were also found at El Mnasra, layer 5, and interpreted as hunting implements^29^. At Dar es-Soltan 1, unit G3-v dated to *c*. 90 ka, a modified rib was interpreted as a “knife-like” tool^30^.

In Central Africa, unilateral barbed points and preforms were found in three MSA sites along the Upper Semliki River at Katanda (Kt) 2, 9 and 16. Kt9 sediments originally yielded optically stimulated luminescence (OSL) ages of *c*. 90 ka^31,32^. More recent dating attempts produced results consistent, albeit scattered, with ages in excess of 60–70 ka, and certainly no younger than 50 ka^33^. The barbed points were made from large mammal ribs and long bone diaphyses shaped by grinding, incising, notching and scraping^34,35^.

In southern Africa, three bone tools were found at Broken Hill (Kabwe) cave, Republic of Zambia, and were interpreted as one projectile point and two “gouges”^36^. The deposit is estimated, based on associated faunal and lithic assemblages, to belong to the late Middle Pleistocene. The “gouges” were modified by marginal scraping. According to the authors, the final shaping of the single point entailed polishing, resulting in the obliteration of traces produced by initial stages of manufacture^36^. A possible bone point is reported from a MSA layer at Mumbwa Cave, also in Zambia^37^, however, see^38^. In Namibia, two notched ribs were found in the Still Bay (SB) layers of Apollo 11 Cave^39^. Three notched pieces from Howiesons Poort (HP) layers at Klasies River, South Africa, are interpreted as tools to work hides or process starchy plants^40,41^. An elongated bone point, worked by scraping, and morphologically similar to those found in the ELSA and Later Stone Age (LSA), comes from an early HP layer at Klasies River^40,42,43^. The distal fragment of a probable projectile point shaped by scraping was securely attributed to a SB layer at Peers Cave, South Africa^40,44^.

Three sites from South Africa, namely Border Cave, Blombos Cave and Sibudu Cave, stand out for the richness and diversity of their bone tool assemblages. At Border Cave, KwaZulu-Natal, notched bones and awls made from split suid tusks worked by scraping and grinding come from layers dated between 60–45 ka. Hunting weapon tips made of bone, a suid tusk spearhead, awls, and a notched baboon fibula, interpreted as a counting device^45^, derive from layers dated 44–40 ka^46^. At Blombos Cave, SB layers dated 75–70 ka have yielded bone spear points shaped by scraping and polishing, tanged points produced by scraping, and thin awls made of mammal and bird bones, transformed by scraping and grinding^40,47^. A single robust point, partially shaped by grinding a weathered bone, comes from the DUN layer overlaying the SB layers^40^ dated to 67.8 ka^48^.

Sibudu Cave is the only African site that has yielded bone tools throughout a stratigraphic sequence dating from *c.* 80–38 ka, including pre-Still Bay (PSB: ~ 80–72 ka), SB (~ 70 ka), HP (65–61 ka), post-HP (PHP: 59–57 ka) and Final MSA (FMSA: 49–38 ka) layers^49,50^. Two wedge-like tools shaped by scraping were recovered from the PSB and HP layers. Awls and a single point are restricted to the HP layers. *Pièces esquillées* (bone fragments marginally modified by scraping and reshaped by grinding and used as splitting tools), bone splinters bearing traces of use, smoothers, and pressure flakers (elongated bone fragments used to retouch stone tools by pressure) come from HP and PHP layers. Two pins were found in HP and FMSA contexts respectively. A few notched pieces are found in PSB, HP and PHP layers^44,46,51^. It has been suggested that the size of the bone point from a HP layer at Sibudu is consistent with its use as an arrow point^44^, a hypothesis supported by micro-focus computed tomography (µCT) analysis of this object and similar experimentally produced and used bone arrow points^52^. Histological analysis of Sibudu bone tools and shaft fragments by means of µCT scans suggests that perissodactyl, artiodactyl, and to a lesser extent primate and carnivore limb bones were used in their manufacture^53^.

**Figure 1:**
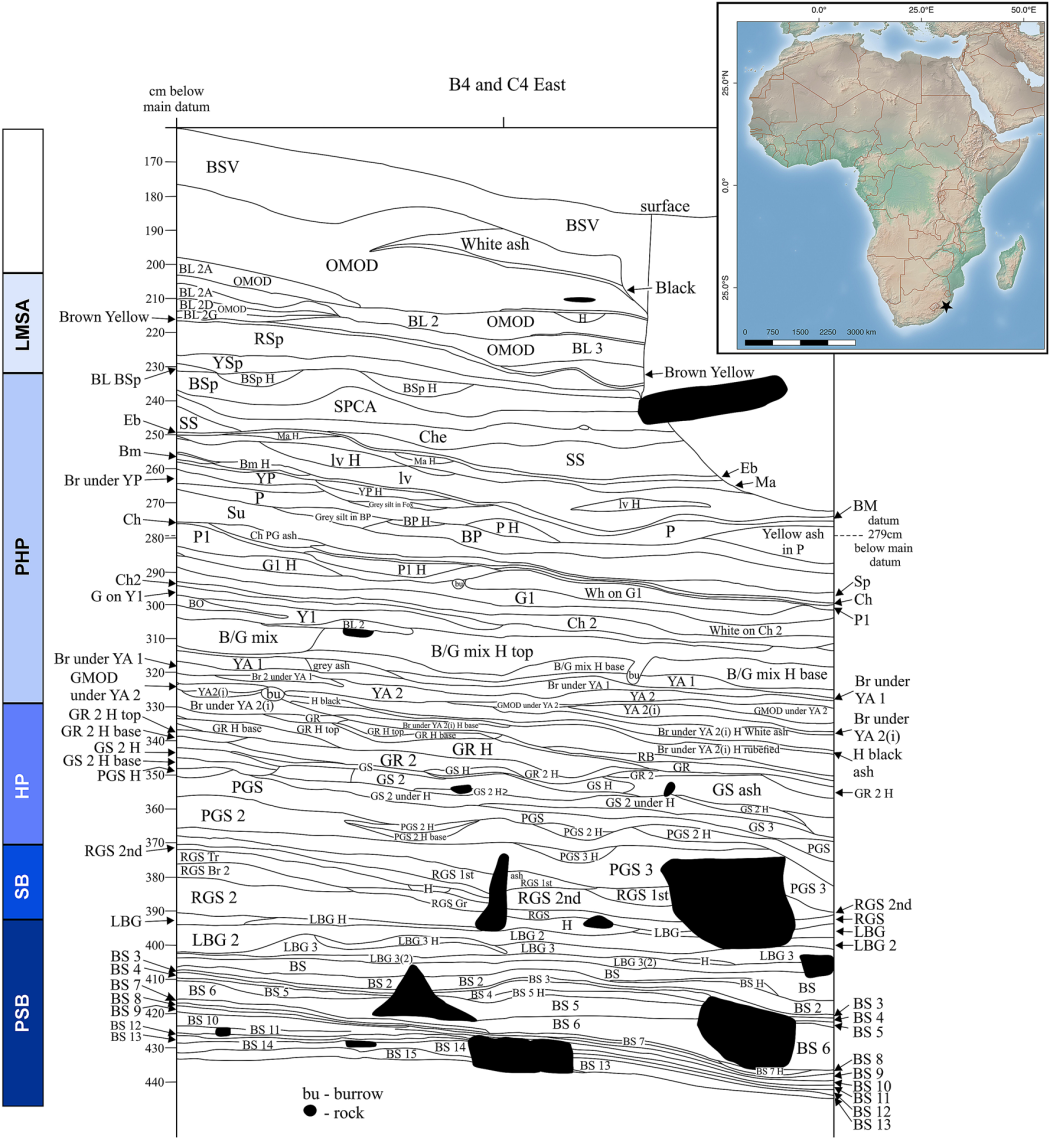
Site location and stratigraphy. Location (star on the map) and stratigraphy of the Sibudu Cave site. The bone tools reported here come from layers attributed to pre-Still Bay (PSB), Still Bay (SB), and Howiesons Poort (HP) technocomplexes (see Table 1). Map insert made by LD using QGIS v. 2.14.3-Essen (Free Software Foundation, Inc., Boston – https://download.qgis.org/downloads/) using free vector and raster from Natural Earth (naturalearthdata.com).

Here we present the findings of technological and functional analyses conducted on an extensive collection of double-beveled bone tools found in PSB, SB and HP layers at Sibudu Cave (Fig. 1). Their number allows for the in-depth documentation of their manufacturing process and morphological variability, as well as investigation of their function(s). With the exception of the barbed points from Katanda, the age of which remains inconclusive, and modified ribs from Northwest Africa, these objects represent one of the earliest well-dated examples of highly standardized bone tools that, unlike the other short-lived occurrences, represent a regional technological tradition that lasted for at least 20,000 years.

Archaeologists have traditionally inferred the function of bone tools through comparative microscopic analysis of use-wear on archaeological artefacts and experimentally used tools^54–58^. Only recently have attempts been made to infer function from the quantitative texture analysis of utilized bone surfaces^59–61^. Experimental work demonstrates that this approach can identify significant differences between bone surfaces subjected to distinct wear processes^61^. The application of this approach to expedient bone tools used by late Neanderthals demonstrated its pertinence in distinguishing natural from anthropogenic use-wear^62^. Other research has explored the application of roughness parameters to distinguish weathering stages recorded on mammal, fish and ostrich ribs^63^. The present study represents the first attempt to infer the function of some of the earliest formal bone tools through the application of texture analysis to archaeological specimens, replicas of the archaeological tools experimentally used in a variety of tasks, and ethnographic artefacts. Our results support a scenario in which, as early as 80,000 years ago, modern human populations linked to specific regions developed and locally maintained highly normative technical innovations for the production and use of formal bone tools.

## Results

**Figure 2:**
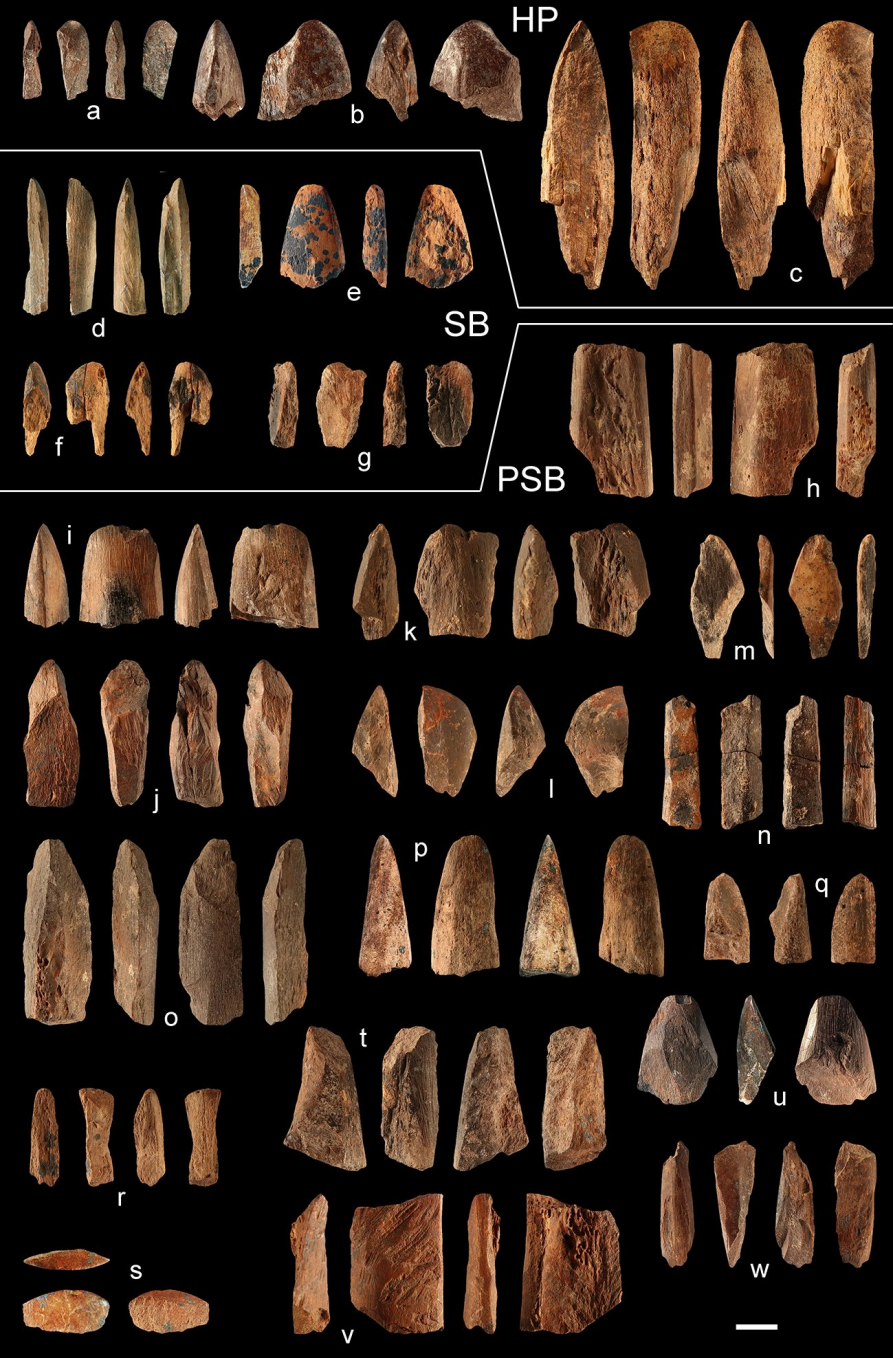
Double-beveled bone tools from Sibudu. The tools were found in Howiesons Poort (**a**–**c**), Still Bay (**d**–**g**) and pre-Still Bay (**h**–**w**) layers. See Table 1 for contextual information, Table S1–S4 for zooarchaeological, morphometric, taphonomic, technological, use-wear, and tip morphometry data. Scale = 1 cm. Photographs by Fd’E.

The twenty-three beveled artefacts come from 15 archaeological layers attributed to three of the five main MSA cultural horizons identified at Sibudu Cave (Fig. 2; Table 1). Sixteen come from PSB layers, four from SB layers and three from HP layers. This sample includes four tools described by Backwell et al.^44^ and d’Errico et al.^46^ but they were not previously subjected to texture analysis.

**Table 1:** Contextual data on the double-beveled bone tools from Sibudu.

	Excavation date	Square	Layer	Layer code	Cultural attribution	OSL (ka)	References
Figures 2(a), 4(a)		B5d	Grey Rocky 2, hearth B	GR2	HP	61.7 ± 2.0	(^45^, Fig. 6)
Figures 2(b), 4(b)	19/11/2009	C4d	Pinkish Grey Sand 2	PGS2	HP	64.7 ± 2.3	(^47^, Fig. 4.6)
Figures 2(c), 4(c), 5(h)	13/11/2009	B4a	Pinkish Grey Sand	PGS3	HP	64.7 ± 2.3	(^47^, Fig. 4.8)
Figures 2(d), 3(d)	11/11/2004	B5b	Reddish Grey Sand	RGS	SB	70.5 ± 2.4	
Figures 2(e), 3(h), 4(e)	11/3/2007	C5d	Reddish Grey Sand	RGS	SB	70.5 ± 2.4	
Figures 2(f), 4(f)	11/3/2007	C5d	Reddish Grey Sand	RGS	SB	70.5 ± 2.4	
Figure 2(g)	16/11/2004	B6a	Reddish Grey Sand 2	RGS2	SB		
Figures 2(h), 3(a)	21/2/2010	B4a	Light Brownish Grey	LBG	PSB	72.5 ± 2.5	
Figures 2(i), 4(i)	21/2/2010	B4b	Light Brownish Grey	LBG	PSB	72.5 ± 2.5	(^47^, Fig. 4.9)
Figures 2(j), 3(e)	17/2/2010	B4d	Light Brownish Grey, under hearth	LBG	PSB	72.5 ± 2.5	
Figure 2(k)	28/2/2010	B4b	Light Brownish Grey 3, hearth 1	LBG3	PSB		
Figures 2(l), 4(l)	28/2/2010	C4d	Light Brownish Grey 3	LBG3	PSB		
Figure 2(m)	31/8/2005	B5a	Brown Sand, Brown Sand 2, hearth 1	BS/BS2	PSB	77.2 ± 2.7	
Figure 2(n)	4/10/2010	B4d	Brown Sand 4	BS4	PSB		
Figure 2(o)	5/10/2010	B4d	Brown Sand 5	BS5	PSB		
Figures 2(p), 3(g), 4(p)	20/10/2010	B4d	Brown Sand 6	BS6	PSB		
Figure 2(q)	5/3/2011	C5b	Brown Sand 7	BS7	PSB		
Figure 2(r)	27/2/2011	C5d	Brown Sand 7	BS7	PSB		
Figures 2(s), 3(i), 5(g)	14/3/2011	C4c	Brown Sand 9	BS9	PSB		
Figures 2(t), 3(b)	14/3/2011	C4c	Brown Sand 9	BS9	PSB		
Figures 2(u), 3(f), 4(u)	25/3/2011	C5d	Brown Sand 12	BS12	PSB		
Figures 2(v), 3(c)	25/3/2011	C5d	Brown Sand 12	BS12	PSB		
Figures 2(w), 5(i)	27/3/2011	B4a	Brown Sand 14	BS14	PSB		

Excavation date, stratigraphic provenance, and cultural attribution of the bone tools reported in the present study. OSL ages (in ka) were reported in^49,55^. Four tools were described in previous publications but were not submitted to texture analysis.

### State of preservation

All of the artefacts are broken and fragmented, none of them retains their proximal portion, and most of them bear longitudinal fractures (Fig. 2; Supplementary Table S1). However, 18 preserve a portion or the entirety of the original bevel, and five are attributed to this tool category based on their general shape and traces of manufacture. A third of the objects bear evidence of heating, probably due to their fortuitous proximity to, or inclusion in, hearths. Thin layers of manganese and calcite deposits are recorded on half of the specimens. Two tools show evidence of having been gnawed by insects, perhaps termites^64,65^. The surfaces not affected by fractures and non-human forms of modification are exceptionally well-preserved, allowing for the study and documentation of anthropogenic modifications.

### Technological analysis

**Figure 3:**
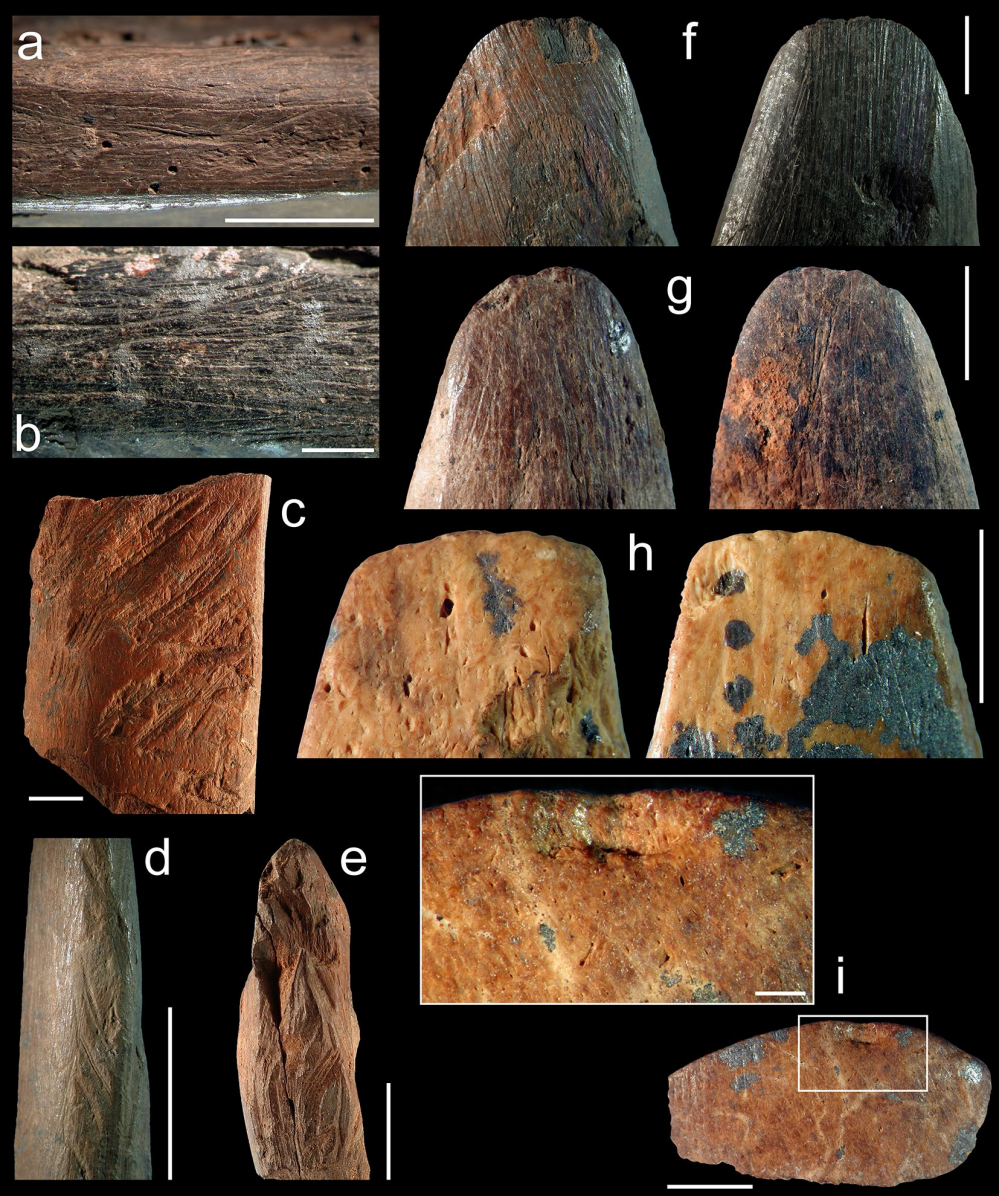
Traces of manufacture and use-wear. The manufacture of the double-beveled bone tools from Sibudu entailed scraping the lateral edges (**a**–**b**), vigorously gouging the flat and lateral sides of the object (**c**–**e**), and shaping the tip by scraping (**f**–**g**). Use-wear takes the form of polish extending over the flat face of the bevel (**g**–**h**) as well as micro-removal scars with polished edges (**i**). Scales: (**b**) = 1 mm; (**a**,**c**–**d**,**f**–**i**) = 5 mm; (**e**) = 1 cm. Photographs by Fd’E.

**Figure 4:**
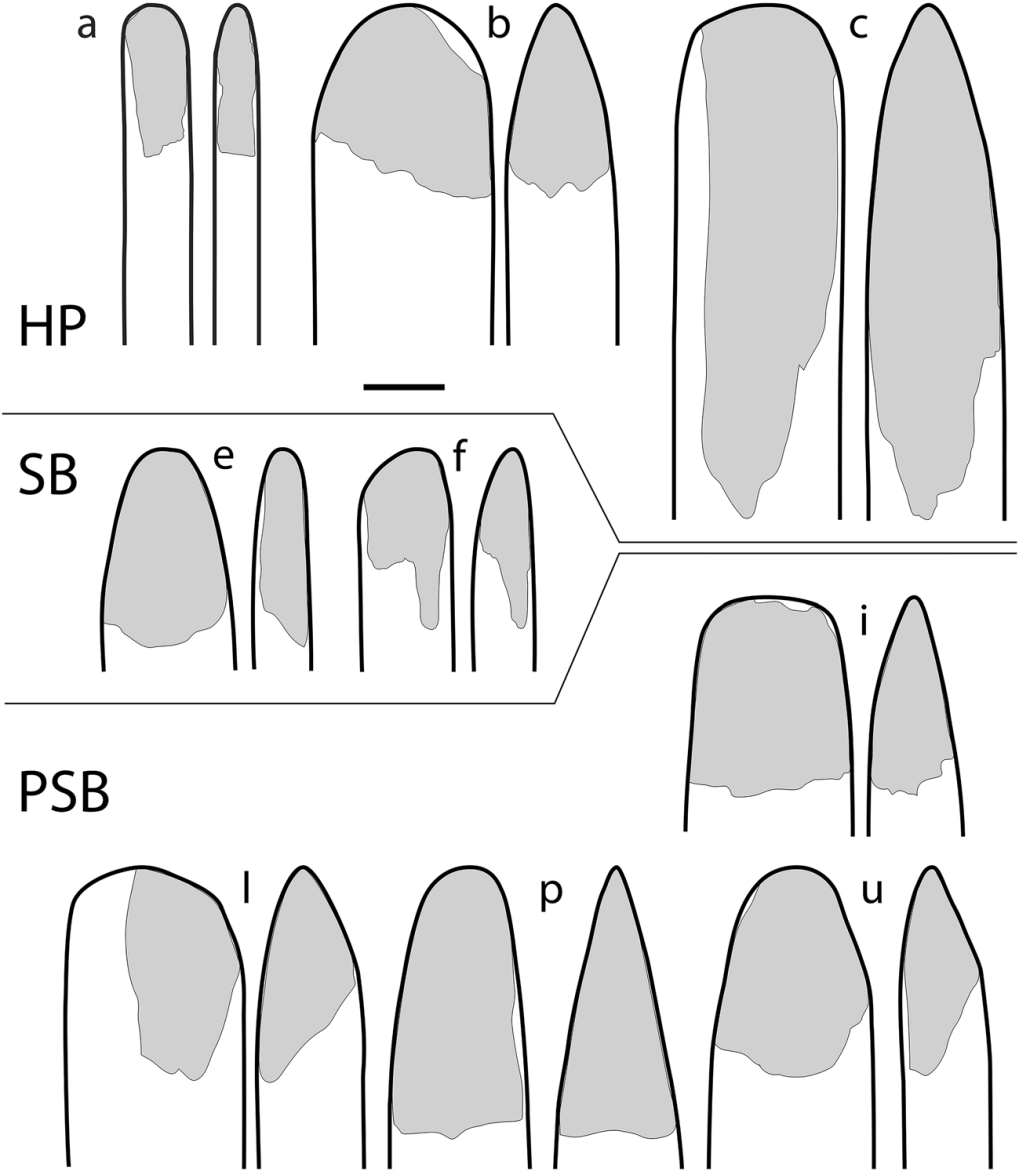
Reconstruction of planar and lateral profiles of the best-preserved double-beveled bone tools from Sibudu**.** The profile reconstruction (thick black outline) is based on complete or near complete specimens found in Howiesons Poort (**a**–**c**), Still Bay (**e**–**f**) and pre-Still Bay (**i**,**l**,**p**,**u**) layers. Grey shaded areas indicate the preserved portion of the tools. Letters match those in Fig. 2 where photographs of the archaeological objects are presented. Scale: = 1 cm.

Apart from a single specimen manufactured from a very large mammal mandible (Fig. 2C), the double-beveled tools from Sibudu were all made on pieces of limb bones. The taxon could not be determined for any of the bone tools. However, archaeozoological analysis, compact bone thickness evaluation, and the study of anthropogenic modifications indicate that the elongated robust shaft fragments (compact bone thickness averaging 8.44 mm, SD: 2.58 mm) derive mainly from medium, large, and very large mammals (Supplementary Table S2), and were shaped by scraping to straighten and flatten their lateral edges (Fig. 3A–B; Supplementary Table S3). Traces of vigorous gouging are recorded on the flat and lateral sides of some of the objects (Figs. 2d,i,j,v, 3c–e). One end was shaped by grinding or scraping to produce a double-beveled rounded edge (Fig. 3f), ogival in lateral section (Fig. 4). On average, the tapering faces meet at an angle of 55.2° (SD: 9.9°; Supplementary Table S4). With the exception of three specimens displaying pristine traces of manufacture and little if any use-wear on their bevels or broad edge (Fig. 2d,p,u), utilization of the beveled end has smoothed manufacturing traces and completely erased them from most specimens, leaving a highly polished, striation-free surface, often extending over the entirety of both flat faces (Fig. 3g–h). Heavily worn beveled edges on seven objects show micro-removal scars polished by wear (Fig. 3i), indicative of damage that occurred during use. Morphometric variation in complete double-beveled tools shows values ranging between 7 and 24 mm in width and between 5 and 11 mm in thickness, with a few broken tools originally displaying a thickness of up to 14 mm (Supplementary Table S2). This shows that Sibudu inhabitants selected robust limb bone shaft fragments to manufacture tools of different size, but with a highly uniform morphology (Fig. 4).

### Texture analysis and functional assessment

**Figure 5:**
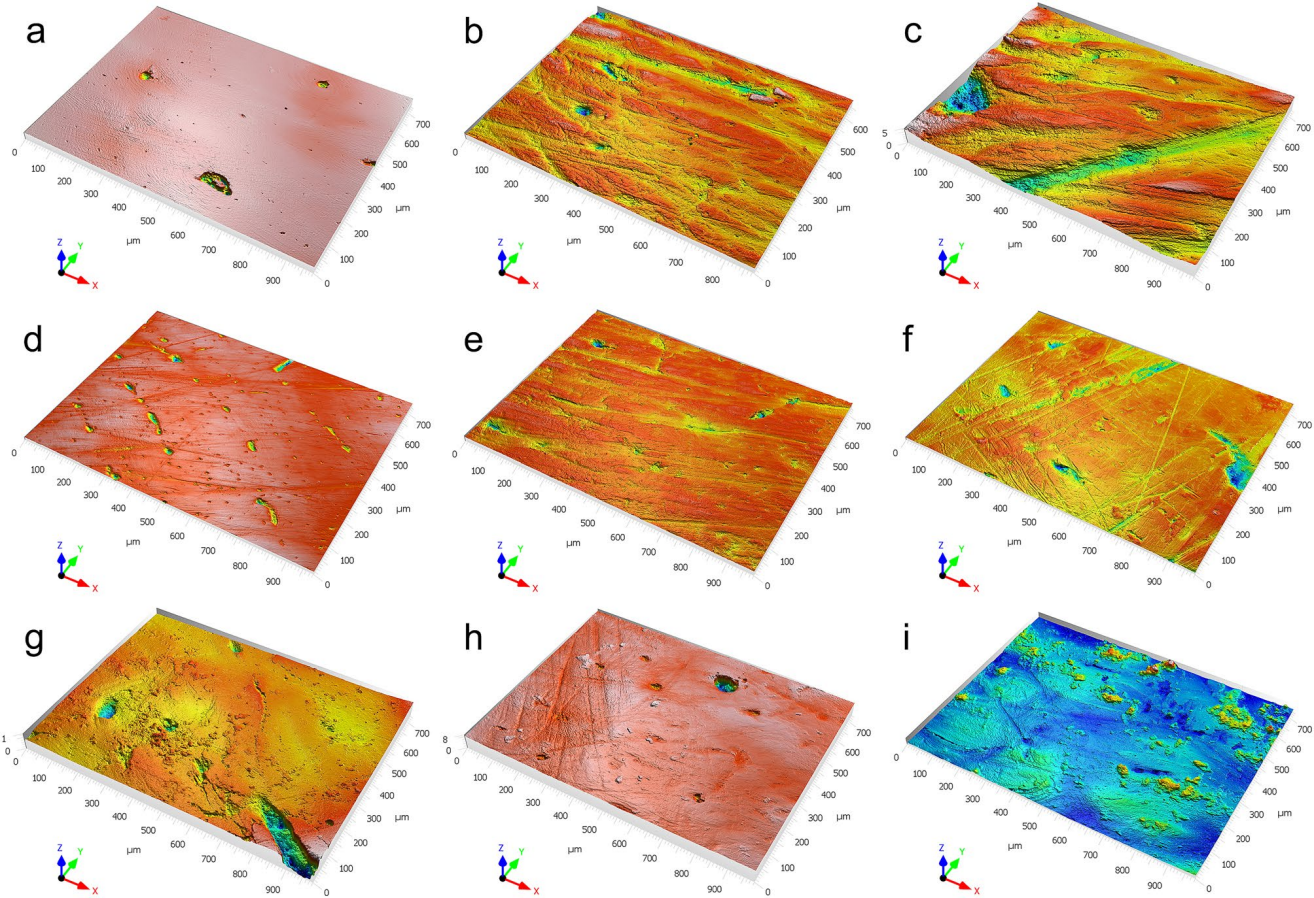
3D renderings of selected areas analyzed by means of texture analysis. Comparison of 3D renderings of unused (**a**) and used (**b**–**i**) tools highlighting variations in wear development and intensity resulting from the use of bone tools in distinct activities, i.e., digging in dry sediment (**b**) versus humus-rich soil for 20 min (**c**), processing rabbit skin without (**d**) and with (**e**) ochre for 20 min, and debarking oak trees (**f**) over an extended period of time. Experimental (**b**–**e**), ethnographic (**f**), and archaeological specimens found in pre-Still Bay layers BS9 and BS14 (see Fig. 2s, w) (**g**,**i**), and in Howiesons Poort layer PGS3 (see Fig. 2c) (**h**).

**Figure 6:**
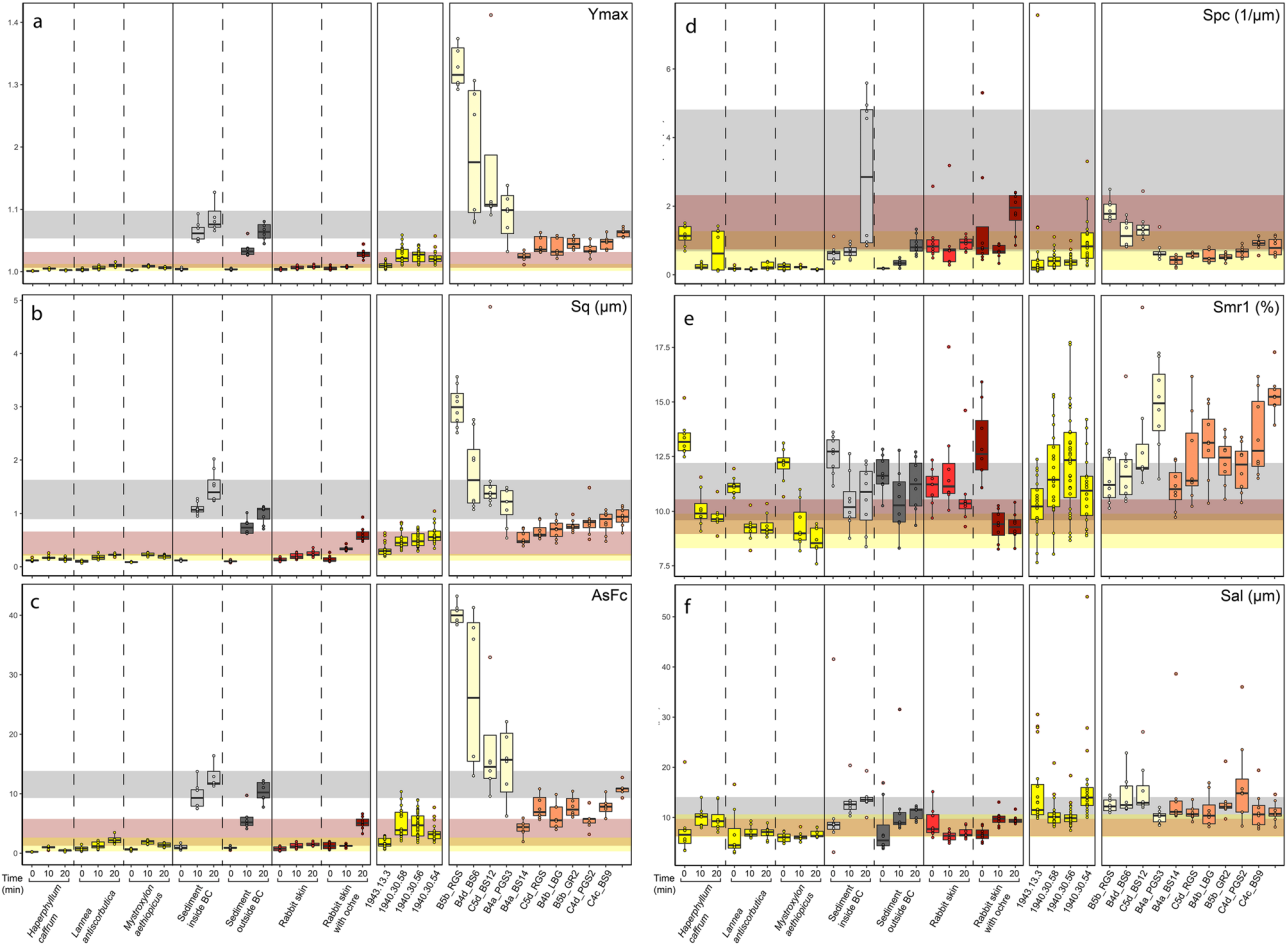
Texture analysis. Boxplots illustrating the variability in textural parameters *Ymax* (**a**), *Sq* (**b**), *AsFc* (**c**), *Spc* (**d**), *Smr1* (**e**), and *Sal* (**f**) on experimental double-beveled tools used, from left to right, in debarking southern African trees, digging in dry sediment and humus-rich soil, and processing rabbit skin with/without ochre, and on ethnographic debarkers as well as on unworn and worn archaeological specimens found at the Sibudu site. Vertical dashed lines separate tools used on different tree species, types of sediment, and presence/absence of ochre mixture for processing rabbit pelt. Numbers on experimental tools indicate length of use in minutes. Horizontal colored bands highlight the range of variability at 1σ for each experimental function (debarking in yellow, digging in sediment in grey, processing pelt in red) after 20 min of use. Mixed colors indicate overlap between ranges. BC = Border Cave.

Evaluation of the acquired data from the six parameters used to analyze surface texture shows substantial similarities and differences between experimental, ethnographic, and worn and unworn archaeological tools (Fig. 5). Three parameters, *Ymax*, *Sq*, and *AsFc*, evaluate the texture as a whole, while *Spc*, *Smr1*, and *Sal* focus on aspects of the surface texture such as peaks (Fig. 6; Supplementary Fig. S1). The former, which in simple terms account for entire surface complexity (*Ymax*), surface homogeneity around the mean height (*Sq*), and surface homogeneity at different scales (*AsFc*), reveal similar trends. First, worn and unworn archaeological specimens show clearly distinct ranges consistent with microscopic observations, indicating that lower values result from the smoothing of the traces of manufacture during tool use. Second, unused experimental tools present comparable roughness values for *Ymax*, *Sq*, and *AsFc*, indicating that the initial states of the working edges were the same regardless of the structure of the limb bone. Third, all experimentally used tools display roughness values higher than those recorded in their unused state. Fourth, tools used to debark trees and process a rabbit pelt without an abrasive product like ochre produce the lowest textural complexity. A higher degree of variability is observed among the wear patterns produced by processing a rabbit pelt tanned with an ochre powder and fat mixture, and on ethnographic bone tools used to debark trees. Tools used for digging in sediment display the highest values among all experimental and ethnographic tools. The values recorded on the tool used to dig in dry sediment on the Border Cave talus slope are consistently higher than those recorded on the tool used to dig in humus-rich soil away from the cave. The range of variation in texture recorded on archaeological tools is wider than that calculated for any of the experimental and ethnographic activities. No overlap is observed between the wear on archaeological tools and that on experimental tools used to debark trees and process rabbit skin without an ochre powder abrasive. Between one and three archaeological tools out of seven display variation compatible with the use-wear produced when experimentally digging humus-rich soil or processing rabbit skin with the ochre and fat compound, and debarking trees as recorded ethnographically. For each parameter, two to three archaeological wear patterns fall outside the range recorded for known activities.

The parameters accounting for the sharpness of the peaks, *Spc*, and the proportion of peak material present above the core surface, *Smr1* (Fig. 6; Supplementary Fig. S1), reveal a clear difference between the wear present on archaeological specimens and that generated by scraping skin covered with an ochre mixture. They show a concurrence between some archaeological wear patterns and those produced experimentally when digging humus-rich soil, as well as those observed on ethnographic debarkers. *Smr1* identifies one archaeological tool bearing a wear pattern that falls outside the experimental and ethnographic ranges. The last parameter, *Sal*, measures the horizontal distance in the direction in which the autocorrelation between slope and distance decreases the fastest. This parameter highlights significant overlap between archaeological, ethnographic, and experimental wear patterns.

**Figure 7:**
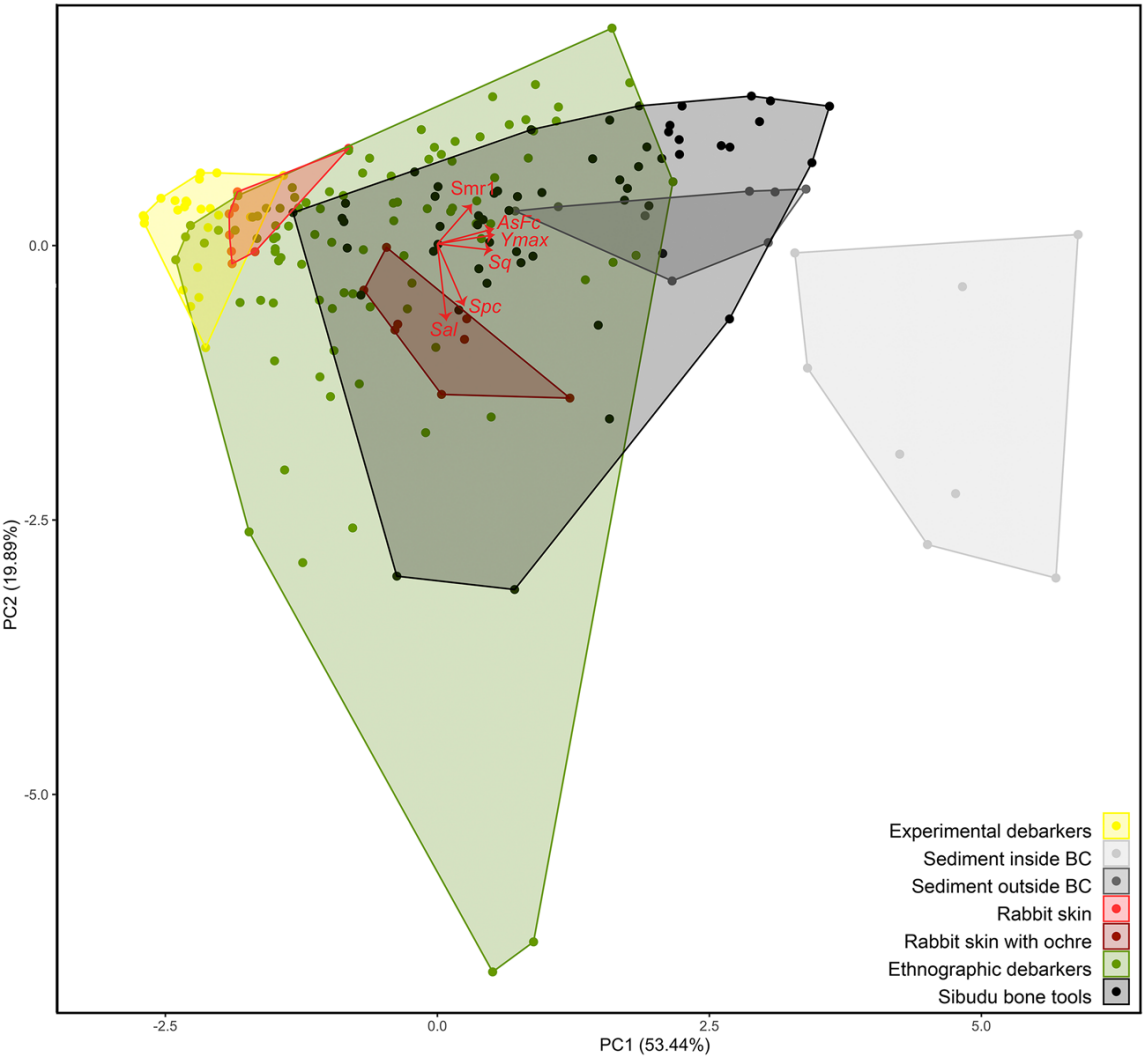
Principal Component Analysis based on six textural parameters. Between-group comparison of the range of variation in textural parameter values on the first two principal components (PC1: 53.44%; PC2: 19.89%). The values recorded on archaeological specimens largely overlap with those measured on ethnographic debarkers, experimental tools used to process rabbit skin with an ochre mixture and to dig in humus-rich soil. BC = Border Cave.

A Principal Component Analysis (PCA) based on the six textural parameters (Fig. 7) reveals that most of the archaeological use-wear patterns overlap with those measured on the ethnographic debarkers, on the experimental tools used to process skin with an ochre mixture, and those used to dig in humus-rich soil. The wear patterns produced experimentally when debarking trees, treating skin without ochre, or digging in dry soil fall outside the convex hull encompassing the archaeological variability. A few archaeological measurements fall outside the range of variation recorded on all experimental and ethnographic tools.

When subjecting experimental and ethnographic textural data to a Flexible Discriminant Analysis (FDA), the model produced in training mode accurately attributes the function for which the tools were used 88.9% on average over 100 iterations; in validation mode, the accuracy is 79.8%. When the 25 best models out of 100 iterations are considered, the validation accuracy rises to 84.3%. This result implies that if the archaeological tools were used for one of the documented functions, the top 25 models would be able to correctly discriminate the function for which they were used 17 times out of 20 (Supplementary Table S5). Application of the two predictive approaches to archaeological use-wear produces similar results: they identify debarking of the kind represented on ethnographic tools as the most likely function for the Sibudu double-beveled tools (Supplementary Table S6). In four instances, tools may have been used to dig in humus-rich soil or for both activities. These predicted functions appear to have been similarly implemented in the three cultural horizons (PSB, SB, HP) in which the archaeological bone tools were found, despite their variation in size.

## Discussion

The double-beveled bone tools discovered at Sibudu in layers dated between ~ 80–60 ka are among the earliest formal bone tools known. Textural and discriminant analyses indicate that most of the tools were probably used in debarking activities that produced use-wear comparable to our ethnographic sample, and possibly for digging in humus-rich soil, likely to extract roots, sedges or underground storage organs. Interestingly, the function of the Sibudu double-beveled tools is not linked to hunting or hide processing activities, with which the production of the first documented formal bone tools, e.g., projectile points, barbed points, smoothers, etc., have been traditionally associated, but rather domestic subsistence strategies devoted to the exploitation of vegetal resources. Extraction of underground plant resources, entailing contact of the tool with both the ground and vegetal matter, may be the source of use-wear patterns with overlapping ranges of values for the recorded textural parameters. Bark from 174 tree species is currently used medicinally in KwaZulu-Natal^66^. Many of these species have been identified amongst the Sibudu charcoal^67,68^ so they may also have been used as firewood. A popular medicinal taxon, *Cryptocarya woodii*, was found on Sibudu sedge bedding, presumably used as an insect repellent^69^, and the poisonous *Spirostachys africana*^70^ bark and wood may have been burned at Sibudu to create insecticidal smoke^68^. San hunter-gatherers use bark from various species for making fiber that is in turn used for construction, binding tools, or making snares. Some roots are excavated and scraped or cut to make traditional glues and adhesives^71^. Extracting and working roots in this way could produce earth-scraping traces and traces emulating bark removal. The differences between the roughness values recorded for the use-wear on experimental and ethnographic debarkers could either be due to differences in the length of utilization of the tools, or differences in the fibrous structure of the bark and sapwood on which the tools were used.

While the discriminant analysis rules out a number of functions, e.g., skin processing with or without ochre, digging in dry sediment, and debarking some tree species, the PCA suggests that some Sibudu wedges were used in activities for which we do not yet have ethnographic or experimental correlates. This is consistent with the tiny size of one or two tools that appear too small for debarking or digging activities. Additional characterization of textural parameters on experimental and ethnographic bone tools must be undertaken to identify the functions for which some of these tools were used. Extension of this research strategy to other bone tools identified in the MSA and the European Middle Paleolithic, and for which a function has been proposed e.g.,^21,22,30^, is necessary to strengthen previous interpretations or identify alternatives or complementary functions.

These results imply that, although many of these tools were apparently used for the same purpose, a double-beveled end was also sought for functions that we are unable to identify at present. It also implies, in light of the stratigraphic distribution of the studied artefacts, that this normative bone tool tradition was transmitted and maintained for at least 20 millennia and associated with three different MSA lithic technological traditions, namely the PSB, SB, and HP. Aside from two morphologically similar tools of uncertain age found at Broken Hill, Zambia, and interpreted as ‘gouges’^36^, none of the numerous MSA sites from South Africa with PSB, SB and HP layers, including those in which well-preserved faunal assemblages and bone tools were found, has yielded double-beveled bone tools similar to those analyzed in this study. In the last three decades, numerous southern African MSA sites have been excavated with modern methods, implying the recovery of small items. Most sequences in caves and shelters that record excellent preservation of organic material, and bone assemblages from new and old excavations at southern African MSA sites have been carefully reexamined by specialists in search of formal and expedient bone tools^36–41,43–47,51–53^. Although old excavation methods and curatorial practices may have affected the recovery of fragmentary bone tools, we now have a clear idea of which types might be present and which archaeological sequences are likely to have yielded such artefacts. In light of this emerging pattern, the Sibudu double-beveled bone tools suggest that behind clear similarities in lithic technology and tool types defining each southern African MSA technocomplex^72^, differences and trends of continuity exist at a regional scale in other aspects of cultural adaptation. This pattern supports, in line with recent research^46,72–79^, a scenario in which regionally distinct MSA human populations developed and maintained localized, lasting technical innovations alongside more ephemeral cultural traits. Finally, our results demonstrate that technological and textural analyses of bone tools are effective means by which to infer human behavior from these artefacts. Furthermore, they contribute to our understanding of where, when and for what purpose innovations like the bone tools emerged and became established in human history.

## Methods

### Archaeological context

The Sibudu site is located on a cliff above the uThongathi River in KwaZulu-Natal, South Africa, about 15 km inland from the Indian Ocean (Fig. 1). Excavations at the site were conducted by one of us (LW) from 1998 to 2011. Since 2011, ongoing excavations have been conducted under the directorship of Nicholas Conard, University of Tübingen. The site features a long and complex stratigraphic sequence comprising more than 50 MSA horizons directly overlain by Iron Age occupations; no LSA occupations are represented at the site. From 1998 to 2011, an area of 21 m^2^ was excavated to a depth of ~ 3 m. The cultural sequence documented during these excavations includes pre-Still Bay, Still Bay, Howiesons Poort, post-Howiesons Poort, late MSA, final MSA and Iron Age technocomplexes^80^. Although complex, the stratigraphy at Sibudu is clear and well-preserved. Geoarchaeological analysis suggests exceptional stratigraphic integrity with minimal vertical mixing between the anthropogenically-formed layers^69,81^. Such integrity can be appreciated from the preservation of laminated, articulated phytoliths and centimeter-thick layers of undisturbed, carbonized bedding, sometimes extending laterally for meters^69^. Disturbance caused by the recent digging of pits, animal burrowing and rockfall was easily recognized and clearly delimited during the excavations.

Sibudu features excellent organic preservation, with bone, charcoal, carbonized seeds and other plant remains found throughout the sequence^80,82,83^. The faunal assemblage from PSB layers is dominated by suids. The oldest PSB deposits also include a diversity of small game^84^. SB and HP layers show a high frequency of blue duiker (*Philantomba monticola*) together with suids and small-medium sized bovids^84,85^. The PHP faunal assemblage is interpreted as reflecting a more open environment with a decrease in small prey and a predominance of large and very large bovids^85^. Raw material such as dolerite and hornfels dominate the lithic assemblages, although quartz and quartzite were also used for manufacturing stone tools, particularly during the HP ^80,86^. The lithic assemblages were subjected to detailed analyses that identified diachronic changes in stone tool technology^87–89^ and the use in the HP and SB of bone pressure flakers to shape stone tools^46,86,88^. A recent chemical characterization of residue present on grindstones found in PSB and SB layers suggests they were used to grind ochre^90^. From 1998 to 2011, more than 9,200 ochre pieces were recovered from the MSA layers^91,92^.

### Sample selection

#### Archaeological sample

The archaeological material analyzed in the present study is curated at the Evolutionary Studies Institute at the University of the Witwatersrand in Johannesburg, South Africa, and comes from the excavations conducted by one of us (LW) at Sibudu (Amafa excavation permit 007/09). Faunal material from these excavations has been systematically analyzed in search of pieces bearing traces of modification. This led to the identification of a variety of bone tools from different archaeological layers described elsewhere^44,46^, as well as our sample comprising 23 complete and broken bone tools featuring one double-beveled end and modified adjacent edges, four of which were described in previous publications^44,46^. Our sample was recovered between 2004 and 2011 from PSB, SB and HP layers (Table 1). On seven tools, a selected portion of the worn area featuring an excellent state of preservation was molded with Coltène President light body dental elastomer (Coltène, Switzerland). Molds were also taken close to the active edge of three supplementary tools with well-preserved traces of scraping, and little if any use-wear.

#### Ethnographic sample

The ethnographic bone tools analyzed in this study, curated at the *Musée des Civilisations de l’Europe et de la Méditerranée* (MuCEM), known until 2005 as *Musée National des Arts et Traditions Populaires* (MNATP) in Marseille, France, were examined at this museum in March 2019. They consist of seven tree bark removers made on horse radii (see Supplementary Fig. S2). The manufacture of these bone tools entailed rounding the prominent areas of the distal epiphysis to ensure adequate grasp while working, and sawing the diaphysis in half at an oblique angle to create a bevel. Hardening of the bone was achieved in some cases by slow-heating the tools in warm ash. An iron blade was inserted in some tools close to the epiphysis^93^. After incising the bark longitudinally with the iron blade or a cutting tool, and then around the trunk at the two ends of the longitudinal incision, the debarker was inserted between the bark and the sapwood—often of oak trees—to detach the bark using pushing and wedging movements. This activity developed a characteristic polish on the beveled area of bone in contact with the bark and sapwood. The debarkers included in our sample were selected for the excellent state of preservation of their active area and the invasive use-wear which has obliterated traces of manufacture. On four tools the worn area was molded with Coltène President light body dental elastomer (Coltène, Switzerland). These tools were used in Umbria, Italy, and various regions of France, particularly Vendée and Loir-et-Cher during the eighteenth and nineteenth century, and were donated to the Museum by Jean Servais, Louis Frenet and Guy Moinet between 1940 and 1950. However, the use of this tool in France dates from the eighth century to the twentieth century, when it was replaced by similarly shaped metal tools^93,94^.

### Technological analysis

Each bone artefact was examined with a Leica Wild M3C stereomicroscope equipped with a Nikon CoolPix 900 digital camera at magnifications ranging from 4–40× and photographed with a Canon PowerShot G7 X Mark II. We have recorded natural and anthropogenic modifications based on criteria established in the literature^2,7,57,95–110^. When possible, we recorded the mammal size class and anatomical origin of the blank, blank extraction and shaping techniques, and traces of use and resharpening. The location and extent of worked areas and the chronology of the technical actions were recorded for each bone artefact. Identification of shaping techniques on archaeological specimens is based on experimental and archaeological data^4,7,14,57,111–116^. Morphometric data were collected with digital calipers and included, when possible, the maximum length, width and thickness, the cortical thickness, and the width and thickness of the beveled area at 5, 10, 15 and 20 mm from the distal end (Supplementary Table S4).

### Experimental design

Seven elongated limb bone fragments of an adult large bovid were transformed into wedge-shaped tools (Supplementary Fig. S3) by shaping their ends through abrasion with an ESCIL 300 GTL lapping and polishing machine using 800-grit paper. The bones used in the experiments were collected from a partially disarticulated eland (*Taurotragus oryx*) skeleton on the farm Spion Kop 932 (28°28′03″S 27°49′05″E), near the town of Senekal in the eastern Free State Province of South Africa. The landscape here is characterized by flat-top mountains of the Karoo Supergroup. Colluvial fans drape the mountain foot-slopes. The host rock of the sedimentary fans is siltstone and sandstone of the Elliot Formation. Many of the fans are dissected by gullies, and the skeleton was found in one of these gullies in a dry riverbed. The bones showed weathering stage 1^110^ with only dry tissue on vertebrae and at the end of long bones, suggesting approximately one year of exposure. The limb bone fragments were selected to match the thickness of most archaeological bone tools. The pieces were dry when shaped. The bevel was polished using a self-adhesive flock polishing cloth (ESCIL, Chassieu, France) covered with a fine diamond solution. The surfaces thus produced were examined with an optical microscope in reflected light, and polishing was repeated until micro-striations were barely detectable at 40× magnification, i.e., striations measuring less than 1 µm in width. Previous research has demonstrated that differences in the original surface texture of bones influence the development of wear^61^. In addition, measuring the development of a use-wear pattern on experimentally used bone tools shaped with prehistoric techniques is challenging because it is difficult to acquire measurements in exactly the same location before and after use. To overcome these issues, our experimental design aimed to produce homogeneous, comparable surfaces on which modifications that developed during experimental use could be precisely located and measured (Supplementary Fig. S4). Textural analysis of the unused active areas confirms that they were characterized by almost identical roughness values (Fig. 6; Supplementary Data S2). This demonstrates that neither the experimental shaping nor the original bone structure influenced roughness parameters prior to the experiments.

Three experimental beveled tools were used to remove sections of bark from three living, endemic, South African trees, namely *Harpephyllum caffrum*, *Lannea antiscorbutica*, and *Mystroxylon aethiopicum. H. caffrum* seeds were found at Sibudu in late MSA layers and *M. aethiopicum* seeds and charcoal throughout the sequence^67,82^. *L*. *antiscorbutica* is traditionally used as a medicinal plant^66,117–121^. Two beveled tools were used as digging tools; one in the ground on the Border Cave (KwaZulu-Natal) talus slope and the other outside the cave. The talus slope floor consists of fine, sorted, abrasive sedimentary particles^122^, while the ground outside is a humus-rich topsoil with sparse gravel supporting grass growth. Two more beveled tools were used to remove fat and connective tissue from two rabbit pelts. After skinning the animals, the pelts were dried for 48 h. One of them was then scraped and cleaned using one of the beveled tools. The other was covered with a mixture of ochre powder and wet-rendered lard and left to dry for an additional 24 h before being scraped with the second beveled tool. All of the experimental tool tips were molded with Coltène President light body dental elastomer (Coltène, Switzerland) before use, and after 10 min and 20 min of use.

### Texture analyses

When necessary, the elastomer molds of the beveled surfaces were cut with a scalpel to expose the worn areas. The resulting samples were analyzed with a Sensofar S Neox confocal microscope equipped with a long-distance 50× lens (numerical aperture = 0.80), allowing a lateral resolution of 0.26 µm and a vertical accuracy of 3 nm. Three-dimensional surface acquisitions were made on both beveled surfaces on an area measuring 0.98 × 0.74 mm located close to the tool’s working edge, i.e., between 2 and 3 mm. Areas displaying anatomical features such as capillaries, and post-depositional damage on the archaeological specimens were avoided as much as possible. Scan quality was reviewed after each acquisition. Scans with 95% or more of the surface measured were retained for further analysis. Less accurate acquisitions were remeasured by improving the light and exposure parameters until 95% of the targeted surface area was acquired.

Post-acquisition surface treatment followed a procedure similar to that described by Martisius et al.^61^, and was performed with Sensomap Mountains 7.4 software. Using built-in operators, it entails levelling the surface with the least square method, mirroring the y- and z- axes to obtain the original surface from molds, removing isolated outliers and those around edges, filling in non-measured points by interpolating from neighbors, and finally removing form using a polynomial of third order. Concavities due to the presence of capillaries and post-depositional damage were manually excluded from the analyzed area. A Gaussian filter of 80 µm was applied as a cut-off to remove waviness from the microtopography. The resulting acquisition was subdivided in four identical quadrants. The following roughness parameters (ISO 25178) were calculated: root mean square height [*Sq*], autocorrelation length [*Sal*], arithmetic mean peak curvature [*Spc*], and upper material ratio [*Smr1*].

Fractal analysis provides an alternative to the measure of roughness. It allows one to document and quantify irregular shapes at multiple scales. In archaeology, this approach was successfully applied to document worn surfaces of bone and stone tools as well as teeth^60,123–134^. The Sensomap Mountains 7.4 software directly analyses the surface and quantifies the fractal parameters [*AsFc*] and [*Ymax*], which respectively correspond to the area-scale fractal complexity and the developed interfacial area ratio, i.e., a parameter equivalent to [*Sdr*] that can be measured on surfaces with missing values. The fractal analysis does not require previous application of a Gaussian filter to remove waviness.

### Statistical analysis

Variation in each textural parameter was documented and compared for experimental, ethnographic, and archaeological specimens. Trends through time in the evolution of this variation were also evaluated for experimental bone tools.

Textural data (Supplementary Data S1) were processed with the ***stats***, ***mda***, ***plyr*** and ***permute*** packages in R-CRAN^135^. Principal Component Analysis was made with the ***stats*** package. The ***mda*** package allows one to perform different types of discriminant analysis. Flexible Discriminant Analysis (FDA) was performed. This extension of linear discriminant analysis uses non-linear combinations of predictors, e.g. splines, and is useful to model multivariate non-normal or non-linear relationships between variables within each group^136^. Every textural parameter gathered from the ethnographic objects and the experimental specimens used for 20 min was included in the analysis. To account for the small sample size of some groups, we performed the FDA in three steps. First, each group was divided into two randomly selected sub-samples, one half for training the model and the other half to validate it. Second, confusion matrices were produced and allowed us to calculate the accuracy of the model for both the training and validating samples. Finally, the model was used to predict in which group the archaeological data were most likely to belong. With ***plyr*** and ***permute***, the three-step FDA was replicated 100 times. The predictions from the 25 models that yielded the highest accuracy values both in training and validation modes were then compared to assess the most likely function of the archaeological specimens.

## Supplementary Information

Supplementary Information 1.

Supplementary Information 2.

## Data Availability

All data are available in the main text or the supplementary materials.
