# Supplementary material for: Technological and functional analysis of 80–60 ka bone wedges from Sibudu (KwaZulu-Natal, South Africa)
Source: Sci Rep. 2022 Sep 29;12:16270. doi: 10.1038/s41598-022-20680-z (PMC9523071; doi:10.1038/s41598-022-20680-z)
Supplement: Supplementary file 1 — Supplementary Information 1. [file 41598_2022_20680_MOESM1_ESM.pdf]

Supplementary information for

Technological and functional analysis of 80-60 ka bone wedges from Sibudu (KwaZulu-Natal, South Africa)

Francesco d'Errico*, Lucinda R. Backwell, Lyn Wadley, Lila Geis, Alain Queffelec, William E. Banks, Luc Doyon

*Corresponding author's email: francesco.derrico@u-bordeaux.fr (Fd'E)

This PDF file includes:

Figs. S1 to S4
Tables S1 to S6

Other Supplementary information for this manuscript includes:
Data S1

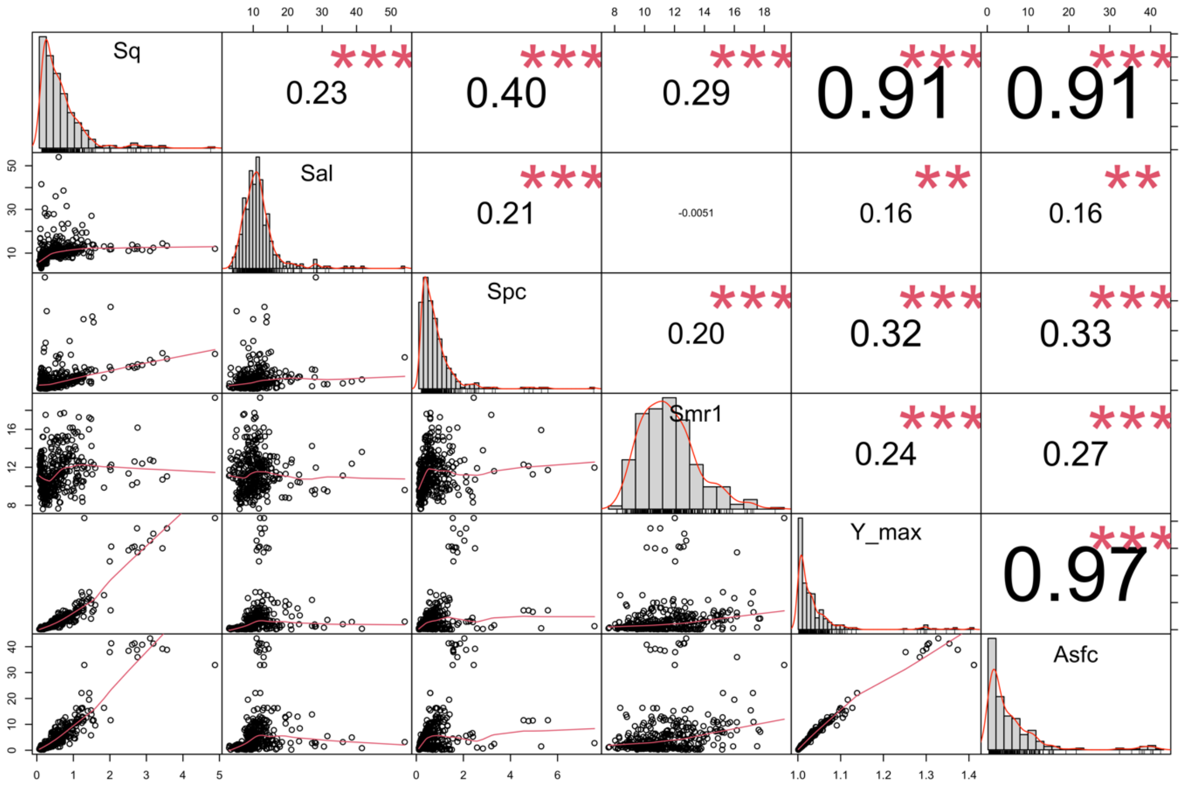

Fig. S1.

Correlation matrix of textural parameters. Below the diagonal, scatterplots and trend lines for the values recorded for pairs of textural parameters. Above the diagonal, R^2 and significance, i.e., **: 0.001 > p ≤ 0.01, ***: p ≤ 0.001, for each pair of textural parameters.

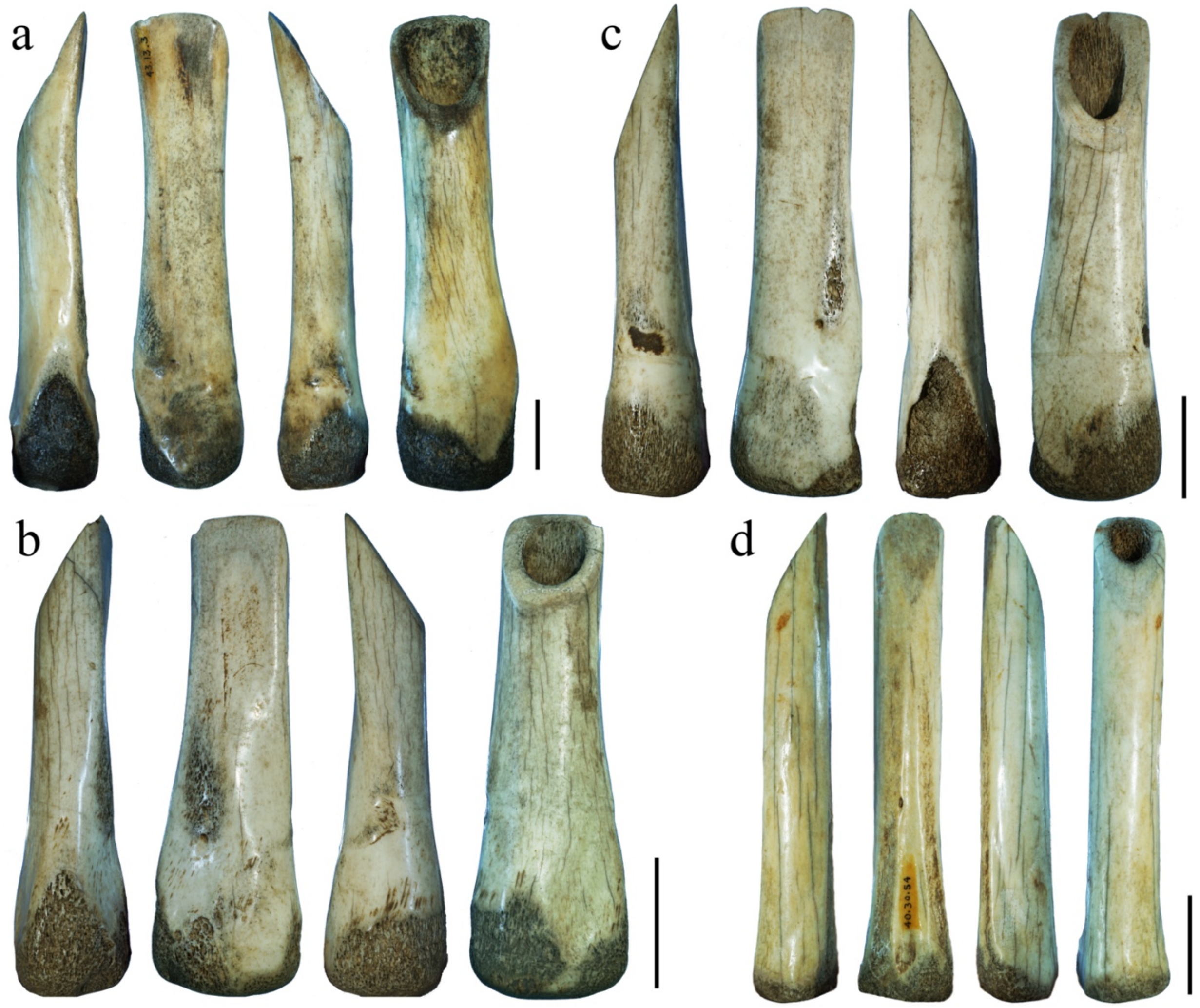

Fig. S2.

Ethnographic debarkers. Bone tools used as debarkers in Italy and France during the 18th and 19th centuries. This sample is curated at the *Musée des civilisations de l'Europe et de la Méditerranée* (MUCEM) under the repository ID 1943.13.3 (a), 1940.30.58 (b), 1940.30.56 (c), and 1940.30.54 (d). Scales = 5 cm. Photographs by LG.

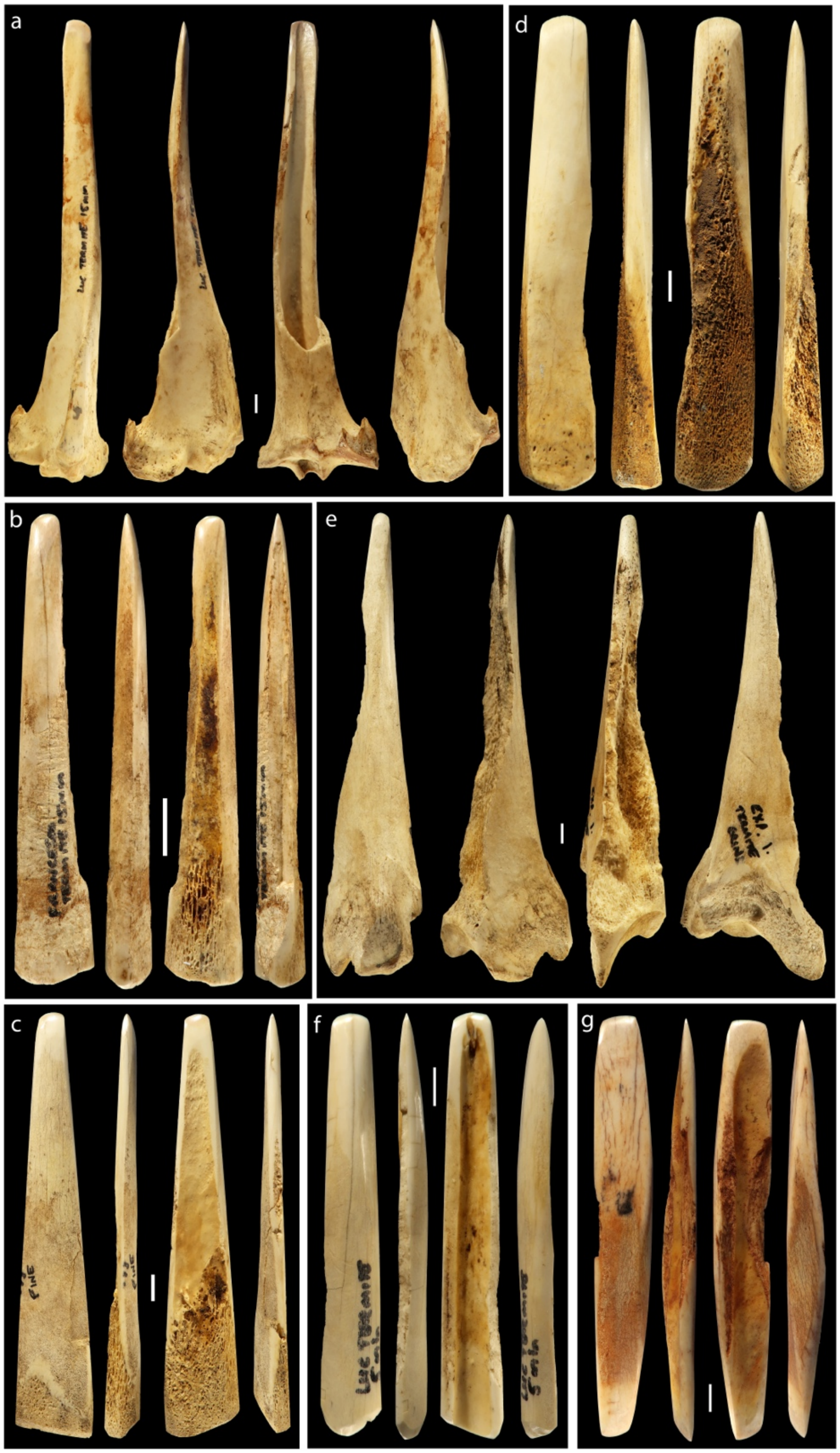

Fig. S3.

Experimental double-beveled bone tools. Bone tools replicated in the present study and used to debark southern African trees (a-c), to dig in dry sediment (d) and humus-rich soil (e), and process rabbit skin without (f) and with an ochre/fat compound (g). Scales = 1 cm. Photographs by Fd'E.

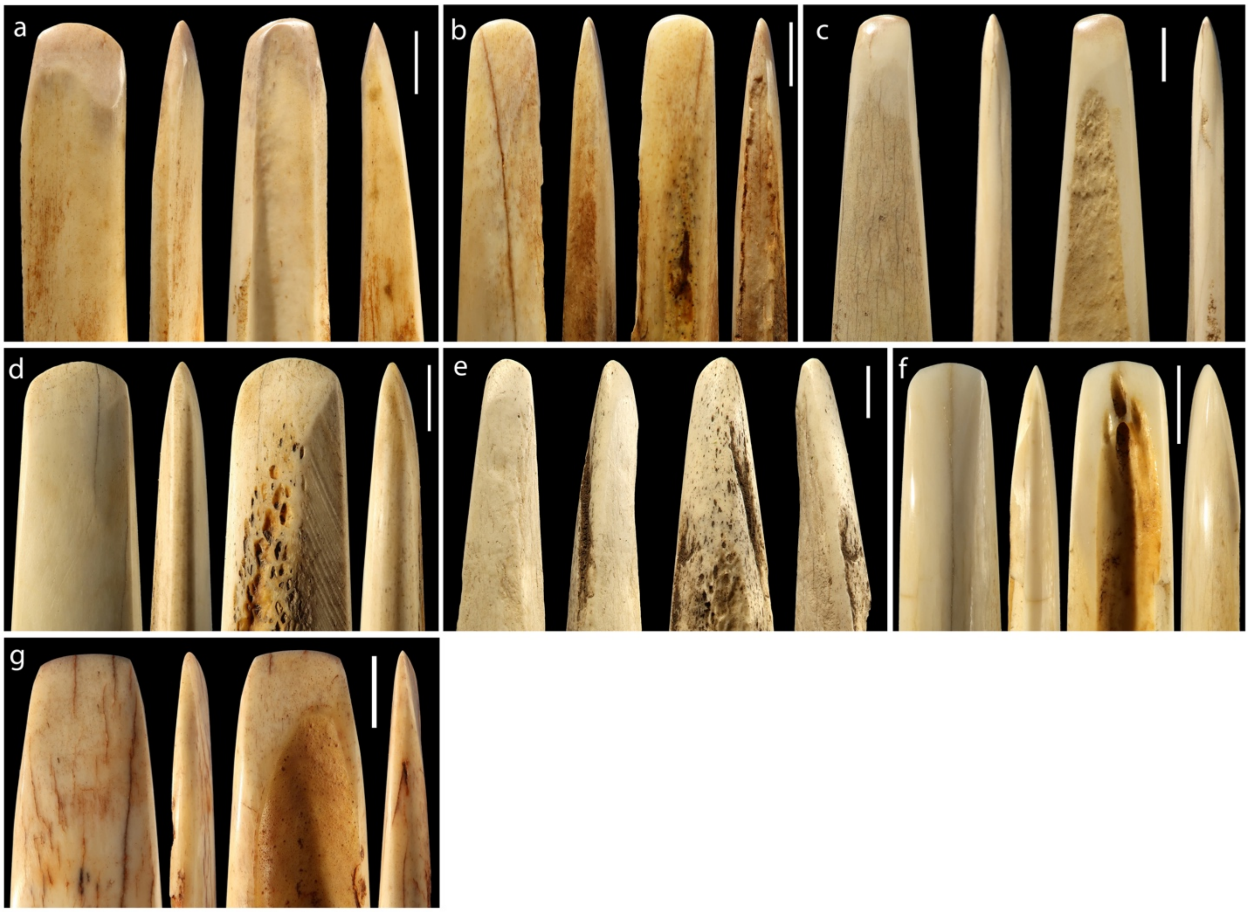

Fig. S4.

Tips of the experimental tools. Close-up view of the tips of the experimental tools used for 20 minutes to debark southern African trees (a-c), to dig in dry sediment (d) and humus-rich soil (e), and process rabbit skin without (f) and with an ochre/fat compound (g). Scales = 1 cm. Photographs by Fd'E.

Table S1.

State of preservation of the double-beveled bone tools from Sibudu. Type and location of the breakage recorded on the Sibudu specimens and non-human modifications and deposits observed on their surfaces. **Breakage:** anc = ancient; flk = flake removal; pdep = post-depositional; spi = spiral; wea = weathered. **Surface modifications and deposits:** con = concretion; heat = exposure to heat; mg = manganese; ter = termite damage.

	Breakage						Surface modifications and deposits
**Figure**	**Proximal**	**Distal**	**Left**	**Right**	**Cortical**	**Medullary cavity**	
Figs. 2(a), 4(a)	anc, wea	none	anc, wea	none	anc, spi	anc, spi	mg
Figs. 2(b), 4(b)	anc, spi	none	none	anc, wea	none	none	mg
Figs. 2(c), 4(c), 5(h)	anc, wea	none	anc, spi	anc, wea	wea, pdep	none	ter
Figs. 2(d), 3(d)	anc, wea	none	anc, wea	none	none	none	mg
Figs. 2(e), 3(h), 4(e)	anc, wea	none	anc, wea	none	anc, wea	indet	mg, ter
Figs. 2(f), 4(f)	pdep	none	pdep	pdep	none	none	mg
Fig. 2(g)	anc, wea	none	anc, wea	anc, wea	none	none	heat
Figs. 2(h), 3(a)	anc, wea	flk	none	partial	none	none	con, ter
Figs. 2(i), 4(i)	pdep	none	none	none	none	none	con, mg, heat
Figs. 2(j), 3(e)	anc, spi	none	anc, spi	none	none	none	mg, heat
Fig. 2(k)	anc, spi	none	anc, spi	none	none	none	con
Figs. 2(l), 4(l)	anc, spi	none	anc, spi	anc, wea	none	none	con, mg
Fig. 2(m)	anc	flk	spi	indet	none	pdep	con, mg, heat
Fig. 2(n)	anc, wea	anc, wea	none	anc, wea	none	none	con, mg, heat
Fig. 2(o)	anc, spi	none	none	none	none	none	con
Figs. 2(p), 3(g), 4(p)	anc, wea	none	none	none	none	none	mg
Fig. 2(q)	anc, wea	none	none	none	none	none	con
Fig. 2(r)	anc, spi	none	anc, wea	anc, wea	none	none	mg
Figs. 2(s), 3(i), 5(g)	anc	flk	anc	anc	none	none	mg
Figs. 2(t), 3(b)	anc, wea	anc, spi	anc, wea	none	none	anc, wea	con, mg, heat
Figs. 2(u), 3(f), 4(u)	anc, spi	none	none	none	anc, spi	anc, spi	con, heat
Figs. 2(v), 3(c)	anc, spi	anc, wea	none	none	none	none	mg
Figs. 2(w), 5(i)	anc	anc, flk	anc	none	none	none	con, mg, heat

Table S2.

Zooarchaeological and morphometric data on the double-beveled bone tools from Sibudu. Data on the mammal size class and skeletal element transformed into tools, and their morphometry. Mammal size class is determined from the compact bone thickness. Indet. = indeterminate; - - indicates that the information could not be measured. Numbers between parentheses are minimal values owing to fragmentation (see Table S1).

Figure	Element	Mammal size class	Compact bone thickness (mm)	Length (mm)	Width (mm)	Thickness (mm)
Figs. 2(a), 4(a)	limb bone	II	(4.58)	(18.58)	7.52	4.59
Figs. 2(b), 4(b)	limb bone	III	10.58	(19.90)	(20.08)	10.81
Figs. 2(c), 4(c), 5(h)	mandible	IV	16.88	(61.35)	(16.61)	13.84
Figs. 2(d), 3(d)	limb bone	I/II	4.17	(30.12)	(6.71)	(6.40)
Figs. 2(e), 3(h), 4(e)	limb bone	indet	5.03	(26.04)	(16.47)	5.57
Figs. 2(f), 4(f)	limb bone	II/III	- -	(24.49)	(11.78)	(6.19)
Fig. 2(g)	limb bone	II	5.36	(20.73)	(11.03)	5.45
Figs. 2(h), 3(a)	limb bone	III	9.38	(39.18)	21.04	9.93
Figs. 2(i), 4(i)	limb bone	II	9.50	(21.45)	18.64	9.50
Figs. 2(j), 3(e)	limb bone	IV	13.04	(13.59)	(11.92)	13.03
Fig. 2(k)	limb bone	II/III	7.37	(24.90)	(17.41)	9.05
Figs. 2(l), 4(l)	limb bone	II/III	9.19	(27.60)	(14.40)	(9.13)
Fig. 2(m)	limb bone	indet	- -	(29.08)	(11.98)	(3.44)
Fig. 2(n)	limb bone	III	7.51	(30.04)	(8.79)	7.51
Fig. 2(o)	limb bone	IV	10.02	(42.26)	15.02	10.27
Figs. 2(p), 3(g), 4(p)	limb bone	II/III	9.49	(26.10)	(15.74)	(9.9)
Fig. 2(q)	limb bone	III	9.53	(19.67)	10.1	9.73
Fig. 2(r)	limb bone	II/III	7.42	(24.54)	(9.04)	7.35
Figs. 2(s), 3(i), 5(g)	limb bone	indet	- -	(10.12)	(18.56)	(4.07)
Figs. 2(t), 3(b)	limb bone	IV	12.53	(32.57)	(16.14)	12.5
Figs. 2(u), 3(f), 4(u)	limb bone	II	- -	(26.55)	(19.04)	(7.45)
Figs. 2(v), 3(c)	limb bone	III	8.17	(33.94)	23.62	8.73
Figs. 2(w), 5(i)	limb bone	II	- -	(30.49)	(10.57)	(7.67)

Table S3.

Technological and use-wear data recorded on the Sibudu double-beveled bone tools. Type and location of manufacturing traces; location, intensity and extent of use-wear; presence of micro-chipping on the tool's active end. Figure numbers with an asterisk indicate specimens subjected to texture analysis. **Technology:** flk = flake removal scars; go = gouging; gr = grinding; ret = retouch; sc = scraping.

	Technology				Use wear					
Figure	Left	Right	Cortical	Medullary cavity	Left	Right	Dorsal	Ventral	Extent	Micro-chipping
Figs. 2(a), 4(a)*		gr		gr		high	high	high	entire	
Figs. 2(b), 4(b)*			sc		high	high	high	high	entire	
Figs. 2(c), 4(c), 5(h)*			sc				high	high	entire	
Figs. 2(d), 3(d)*		sc, go	sc, gr	sc, gr		low	none	low		
Figs. 2(e), 3(h), 4(e)*		gr	gr	gr		moderate	high	high		
Figs. 2(f), 4(f)			sc	sc			low	low		
Fig. 2(g)			sc	sc	low		low	moderate	marginal	
Figs. 2(h), 3(a)	sc	sc		sc						
Figs. 2(i), 4(i)*	sc	sc	sc	sc, go	high	moderate	high	high	entire	Yes
Figs. 2(j), 3(e)		sc	sc, go	sc	high	high	high	high	entire	Yes
Fig. 2(k)		sc	sc	sc/gr	high	moderate	moderate	high	entire	
Figs. 2(l), 4(l)			sc		high		high	high	entire	
Fig. 2(m)			sc	n	high		high			Yes
Fig. 2(n)	sc						low	low		
Fig. 2(o)			flk							Yes
Figs. 2(p), 3(g), 4(p)*	sc	sc	sc	sc	low		low	low		
Fig. 2(q)				ret						
Fig. 2(r)			sc	sc, gr			moderate	moderate	marginal	Yes
Figs. 2(s), 3(i), 5(g)*			sc	sc			high	high	entire	Yes
Figs. 2(t), 3(b)		sc				moderate	moderate	moderate		
Figs. 2(u), 3(f), 4(u)*	sc	sc	sc	sc	low	low	low	low	entire	Yes
Figs. 2(v), 3(c)		sc	sc, go				low			
Figs. 2(w), 5(i)*		sc	sc	sc		moderate	moderate	high		

Table S4.

Morphometric variability of the active edge of the double-beveled bone tools from Sibudu. Width and thickness (in mm) of the active end recorded at 5, 10, 15, and 20 mm from the tip. Cells with "- -" indicate the information could not be recorded due to breakage.

	Thickness (mm)				Width (mm)			
**Figure**	**@ 5 mm**	**@ 10 mm**	**@ 15 mm**	**@ 20 mm**	**@ 5 mm**	**@ 10 mm**	**@ 15 mm**	**@ 20 mm**
Figs. 2(a), 4(a)	4.34	- -	- -	- -	- -	- -	- -	- -
Figs. 2(b), 4(b)	6.36	9.27	- -	- -	14.31	18.36	- -	- -
Figs. 2(c), 4(c), 5(h)	6.77	9.90	12.22	12.54	14.35	- -	- -	- -
Figs. 2(d), 3(d)	3.52	4.01	4.40	4.58	- -	- -	- -	- -
Figs. 2(e), 3(h), 4(e)	3.74	5.27	5.08	- -	- -	- -	- -	- -
Figs. 2(f), 4(f)	5.59	7.03	- -	- -	- -	- -	- -	- -
Fig. 2(g)	3.67	4.70	4.96	- -	- -	- -	- -	- -
Figs. 2(i), 4(i)	4.59	7.05	8.86	- -	16.23	17.42	18.27	- -
Figs. 2(j), 3(e)	6.94	9.13	10.98	12.80	8.64	11.52	- -	- -
Fig. 2(k)	5.53	7.14	7.48	- -	- -	- -	- -	- -
Figs. 2(l), 4(l)	4.43	7.65	9.31	- -	- -	- -	- -	- -
Figs. 2(p), 3(g), 4(p)	4.63	6.70	8.47	9.48	9.53	11.46	12.49	12.48
Fig. 2(r)	5.55	6.60	6.83	6.98	- -	- -	- -	- -
Figs. 2(s), 3(i), 5(g)	4.36	- -	- -	- -	- -	- -	- -	- -
Figs. 2(u), 3(f), 4(u)	4.36	6.77	- -	- -	10.79	14.42	16.52	- -

Table S5.

Flexible Discriminant Analysis (FDA) accuracy of actual versus predicted tool function. Confusion matrix contrasting the actual versus predicted function of tools based on their textural data after 100 iterations (top) and for the top 25 models with the highest accuracy both in training and validation mode (bottom). BC = Border Cave.

100 Iterations							
		Actual					
		Ethnographic debarkers	Experimental debarkers	Sediment inside BC	Soil outside BC	Rabbit skin untreated	Rabbit skin with ochre
Predicted	Ethnographic debarkers	5812	102	0	11	39	97
	Experimental debarkers	143	1042	0	0	256	1
	Sediment inside BC	0	0	340	1	0	0
	Sediment outside BC	124	0	58	383	0	0
	Rabbit skin	33	56	0	0	105	0
	Rabbit skin with ochre	88	0	2	5	0	302

Top 25							
		Actual					
		Ethnographic debarkers	Experimental debarkers	Sediment inside BC	Soil outside BC	Rabbit skin untreated	Rabbit skin with ochre
Predicted	Ethnographic debarkers	1448	32	0	0	11	32
	Experimental debarkers	31	256	0	0	63	0
	Sediment inside BC	0	0	86	0	0	0
	Sediment outside BC	41	0	14	98	0	1
	Rabbit skin	10	12	0	0	26	0
	Rabbit skin with ochre	20	0	0	2	0	67

Table S6.

Flexible Discriminant Analysis (FDA) conducted on the double-beveled bone tools from Sibudu. Probability that the use-wear measured on each analyzed archaeological tool is attributed to a specific function after 100 iterations (left) of the predictive model and its validation, as well as for the top 25 models (right) that obtained the best accuracy scores both in training and validation modes. BC = Border Cave.

		100 iterations						Top 25 models					
Cultural attribution	Contextual information	Ethnographic debarkers	Experimental debarkers	Sediment inside BC	Soil outside BC	Rabbit skin untreated	Rabbit skin with ochre	Ethnographic debarkers	Experimental debarkers	Sediment inside BC	Soil outside BC	Rabbit skin untreated	Rabbit skin with ochre
HP	B5b_GR2	76.75%	0.00%	1.25%	21.88%	0.00%	0.13%	76.00%	0.00%	2.00%	22.00%	0.00%	0.00%
HP	C4d_PGS2	79.63%	0.00%	0.00%	20.38%	0.00%	0.00%	79.00%	0.00%	0.00%	21.00%	0.00%	0.00%
HP	B4a_PGS3	100.00%	0.00%	0.00%	0.00%	0.00%	0.00%	100.00%	0.00%	0.00%	0.00%	0.00%	0.00%
SB	C5d_RGS	90.00%	0.00%	0.00%	8.50%	1.50%	0.00%	90.50%	0.00%	0.00%	8.50%	1.00%	0.00%
PSB	B4b_LBG	80.75%	0.00%	0.00%	19.25%	0.00%	0.00%	77.50%	0.00%	0.00%	22.50%	0.00%	0.00%
PSB	C4c_BS9	47.50%	0.00%	0.00%	52.50%	0.00%	0.00%	44.00%	0.00%	0.00%	56.00%	0.00%	0.00%
PSB	B4a_BS14	92.75%	0.00%	0.00%	7.25%	0.00%	0.00%	91.50%	0.00%	0.00%	8.50%	0.00%	0.00%

Data S1. (separate file)

Textural data. Values obtained when calculating the textural parameters on archaeological, experimental and ethnographic bone tools (ISO 25178) *Sq*, *Sal*, *Spc*, *Smr1*, *Ymax*, and *AsFc*.
