# Supplementary material for: Technological and functional analysis of 80–60 ka bone wedges from Sibudu (KwaZulu-Natal, South Africa)
Source: Sci Rep. 2022 Sep 29;12:16270. doi: 10.1038/s41598-022-20680-z (PMC9523071; doi:10.1038/s41598-022-20680-z)
Supplement: Supplementary file 2 — Supplementary Information 2. [file 41598_2022_20680_MOESM2_ESM.pdf]

Specimen ID	Context	Worked material	Time used (min)	Area_ID	Sq (µm)	Sal (µm)	Spc (1/µm)	Smr1 (%)	Y_max	Asfc
Harpephyllum_unused	Experimental	- -	0	1	0.123807177	7.923672449	0.692756839	12.76912065	1.000907828	0.183807312
Harpephyllum_unused	Experimental	- -	0	2	0.099997814	21.06289713	1.079564388	12.48332895	1.000933544	0.213843728
Harpephyllum_unused	Experimental	- -	0	3	0.105051029	5.293197622	0.825646364	12.74243454	1.000933544	0.213843728
Harpephyllum_unused	Experimental	- -	0	4	0.176146707	7.512300862	1.397601481	15.18528609	1.001233654	0.256920647
Harpephyllum_unused	Experimental	- -	0	5	0.110488645	7.409940801	1.187621688	13.3587012	1.001373016	0.265161801
Harpephyllum_unused	Experimental	- -	0	6	0.126009609	3.49735821	1.510254908	13.69972288	1.000962805	0.235290539
Harpephyllum_unused	Experimental	- -	0	7	0.118112632	5.723550798	1.431419167	13.5395972	1.000962805	0.235290539
Harpephyllum_unused	Experimental	- -	0	8	0.12738459	3.410726059	1.070749197	12.97296732	1.001040509	0.219866935
Harpephyllum_10min	Experimental	Tree bark (*Harpephyllum* sp.)	10	1	0.159571166	11.24567972	0.174773766	9.44266343	1.005744598	1.067178905
Harpephyllum_10min	Experimental	Tree bark (*Harpephyllum* sp.)	10	2	0.259391277	7.957611735	0.187761047	11.09951641	1.004668362	0.995378781
Harpephyllum_10min	Experimental	Tree bark (*Harpephyllum* sp.)	10	3	0.161736776	8.487610988	0.195564958	9.743451648	1.004668362	0.995378781
Harpephyllum_10min	Experimental	Tree bark (*Harpephyllum* sp.)	10	4	0.169687051	10.58088318	0.186580068	9.090428113	1.005145903	1.077470681
Harpephyllum_10min	Experimental	Tree bark (*Harpephyllum* sp.)	10	5	0.164697085	8.328911712	0.387653883	9.764472737	1.002470315	0.498855074
Harpephyllum_10min	Experimental	Tree bark (*Harpephyllum* sp.)	10	6	0.149561147	10.21385879	0.23072662	10.03506951	1.006225292	1.201844847
Harpephyllum_10min	Experimental	Tree bark (*Harpephyllum* sp.)	10	7	0.208902381	10.13044885	0.323317129	11.55115866	1.006225292	1.201844847
Harpephyllum_10min	Experimental	Tree bark (*Harpephyllum* sp.)	10	8	0.138630568	14.0084869	0.286996342	10.10972249	1.002904419	0.604048649
Harpephyllum_20min	Experimental	Tree bark (*Harpephyllum* sp.)	20	1	0.128381006	9.449717548	1.26691	9.546851905	1.002037145	0.420049119
Harpephyllum_20min	Experimental	Tree bark (*Harpephyllum* sp.)	20	2	0.151674639	13.74347764	1.095568331	9.854094642	1.002686782	0.552224334
Harpephyllum_20min	Experimental	Tree bark (*Harpephyllum* sp.)	20	3	0.126284879	8.26231579	1.420512068	9.671533528	1.002686782	0.552224334
Harpephyllum_20min	Experimental	Tree bark (*Harpephyllum* sp.)	20	4	0.134992903	7.088375137	1.288457002	9.939621525	1.002483112	0.503392595
Harpephyllum_20min	Experimental	Tree bark (*Harpephyllum* sp.)	20	5	0.188204105	9.554539847	0.144875965	10.89991806	1.001181756	0.212026339
Harpephyllum_20min	Experimental	Tree bark (*Harpephyllum* sp.)	20	6	0.104565577	7.754620429	0.12466138	8.864019845	1.003265178	0.527496388
Harpephyllum_20min	Experimental	Tree bark (*Harpephyllum* sp.)	20	7	0.20265671	13.93393767	0.141512747	9.601245961	1.003265178	0.527496388
Harpephyllum_20min	Experimental	Tree bark (*Harpephyllum* sp.)	20	8	0.099811023	9.10019084	0.110413539	9.498081407	1.001213514	0.222479598
Lannea_unused	Experimental	- -	0	1	0.119631775	2.976894997	0.284619594	11.10152196	1.003682672	0.901579197
Lannea_unused	Experimental	- -	0	2	0.104034967	4.352787054	0.192236631	11.9425094	1.006122938	1.485457271
Lannea_unused	Experimental	- -	0	3	0.123080903	3.243076364	0.202167526	11.19616076	1.006122938	1.485457271
Lannea_unused	Experimental	- -	0	4	0.12328312	4.601456983	0.205493044	11.166382	1.004039835	0.905215935
Lannea_unused	Experimental	- -	0	5	0.090881728	16.5955267	0.158367767	11.49720987	1.001304588	0.321372517
Lannea_unused	Experimental	- -	0	6	0.067436348	11.62972343	0.135172823	10.83283383	1.001755876	0.429982153
Lannea_unused	Experimental	- -	0	7	0.066992664	6.535786996	0.156949859	10.62033342	1.001755876	0.429982153
Lannea_unused	Experimental	- -	0	8	0.099953445	4.112366772	0.140527939	10.8588648	1.002756143	0.638284725
Lannea_10min	Experimental	Tree bark (*Lannea* sp.)	10	1	0.139118227	6.629186173	0.147879359	8.200472624	1.00277917	0.573646799
Lannea_10min	Experimental	Tree bark (*Lannea* sp.)	10	2	0.133762858	7.330854344	0.127690936	9.09056843	1.00444662	0.967359036
Lannea_10min	Experimental	Tree bark (*Lannea* sp.)	10	3	0.151066564	8.694785895	0.163325142	9.242055143	1.00444662	0.967359036
Lannea_10min	Experimental	Tree bark (*Lannea* sp.)	10	4	0.127768053	6.632481575	0.144233707	9.477825629	1.003242083	0.686920828
Lannea_10min	Experimental	Tree bark (*Lannea* sp.)	10	5	0.266807081	6.374954037	0.173166441	8.76507374	1.006586905	1.385142217
Lannea_10min	Experimental	Tree bark (*Lannea* sp.)	10	6	0.197959984	6.080443668	0.161445947	9.392300169	1.010585237	2.136923858
Lannea_10min	Experimental	Tree bark (*Lannea* sp.)	10	7	0.270049956	9.327836946	0.203169656	10.28347484	1.010585237	2.136923858
Lannea_10min	Experimental	Tree bark (*Lannea* sp.)	10	8	0.197042057	5.917826058	0.173335393	9.300634964	1.008149015	1.822801526
Lannea_20min	Experimental	Tree bark (*Lannea* sp.)	20	1	0.232721672	7.65316353	0.381354099	8.818456167	1.008606373	1.8450033
Lannea_20min	Experimental	Tree bark (*Lannea* sp.)	20	2	0.220037973	8.941609539	0.164540006	8.965089613	1.015856074	3.485077054
Lannea_20min	Experimental	Tree bark (*Lannea* sp.)	20	3	0.260594105	6.684168369	0.38607236	10.35463274	1.015856074	3.485077054
Lannea_20min	Experimental	Tree bark (*Lannea* sp.)	20	4	0.222956824	7.524320276	0.20844787	9.013535944	1.009425033	2.073365604
Lannea_20min	Experimental	Tree bark (*Lannea* sp.)	20	5	0.233161001	5.104662125	0.21792186	9.321484533	1.007486597	1.602189369
Lannea_20min	Experimental	Tree bark (*Lannea* sp.)	20	6	0.197358137	7.491627973	0.365704132	9.110115504	1.01051236	2.291013396
Lannea_20min	Experimental	Tree bark (*Lannea* sp.)	20	7	0.235391075	5.420003984	0.171511426	9.16528808	1.01051236	2.291013396
Lannea_20min	Experimental	Tree bark (*Lannea* sp.)	20	8	0.180981939	6.738858541	0.153144311	9.91803949	1.006856436	1.530801541
Mystroxylon_unused	Experimental	- -	0	1	0.095216229	7.311169914	0.143652669	11.38227403	1.001564914	0.329545482
Mystroxylon_unused	Experimental	- -	0	2	0.079478809	6.335487543	0.123565304	12.31004608	1.002277205	0.540181803
Mystroxylon_unused	Experimental	- -	0	3	0.091752532	7.208824894	0.147101109	10.66755623	1.002277205	0.540181803
Mystroxylon_unused	Experimental	- -	0	4	0.096645478	5.773641055	0.174571744	12.24359813	1.002231642	0.485238946
Mystroxylon_unused	Experimental	- -	0	5	0.087215097	5.30907246	0.329303299	13.11596987	1.00297046	0.73150168
Mystroxylon_unused	Experimental	- -	0	6	0.08629172	4.906640925	0.295146327	12.81369195	1.003294199	0.8308394
Mystroxylon_unused	Experimental	- -	0	7	0.083339252	4.815246716	0.320385762	12.10560164	1.003294199	0.8308394
Mystroxylon_unused	Experimental	- -	0	8	0.081772858	6.537881867	0.311672199	12.22364268	1.002653889	0.682236237
Mystroxylon_10min	Experimental	Tree bark (*Mystroxylon* sp.)	10	1	0.255923681	6.196653839	0.183355601	9.734508502	1.007518406	1.464558709
Mystroxylon_10min	Experimental	Tree bark (*Mystroxylon* sp.)	10	2	0.270546611	6.763820378	0.179272252	10.6394743	1.007152323	1.535072994
Mystroxylon_10min	Experimental	Tree bark (*Mystroxylon* sp.)	10	3	0.215925037	6.026703392	0.24932023	9.033035828	1.007152323	1.535072994
Mystroxylon_10min	Experimental	Tree bark (*Mystroxylon* sp.)	10	4	0.260159361	8.164850545	0.297833696	11.00422356	1.011095298	1.949386113
Mystroxylon_10min	Experimental	Tree bark (*Mystroxylon* sp.)	10	5	0.216459433	5.689736398	0.236000403	8.943126789	1.008856447	1.984935714
Mystroxylon_10min	Experimental	Tree bark (*Mystroxylon* sp.)	10	6	0.205013444	6.182700361	0.222005954	8.185397924	1.01014514	2.281780757
Mystroxylon_10min	Experimental	Tree bark (*Mystroxylon* sp.)	10	7	0.210656513	5.788617029	0.235951052	8.60095609	1.01014514	2.281780757
Mystroxylon_10min	Experimental	Tree bark (*Mystroxylon* sp.)	10	8	0.184271437	5.099033641	0.2088679	8.706137251	1.008834198	2.064215105
Mystroxylon_20min	Experimental	Tree bark (*Mystroxylon* sp.)	20	1	0.235991952	6.177047178	0.140506985	8.325712515	1.00766417	1.707713499
Mystroxylon_20min	Experimental	Tree bark (*Mystroxylon* sp.)	20	2	0.213222672	6.259662849	0.174292926	8.382884698	1.008228677	1.783255117
Mystroxylon_20min	Experimental	Tree bark (*Mystroxylon* sp.)	20	3	0.223113768	6.387467915	0.129537846	8.306556326	1.008228677	1.783255117
Mystroxylon_20min	Experimental	Tree bark (*Mystroxylon* sp.)	20	4	0.208924614	8.093675563	0.151936563	9.295675768	1.007752004	1.693853549
Mystroxylon_20min	Experimental	Tree bark (*Mystroxylon* sp.)	20	5	0.199338034	7.012599939	0.155979893	9.209875801	1.004448168	0.935489775
Mystroxylon_20min	Experimental	Tree bark (*Mystroxylon* sp.)	20	6	0.16043796	8.047343343	0.17162305	9.433647511	1.004884579	1.078257373
Mystroxylon_20min	Experimental	Tree bark (*Mystroxylon* sp.)	20	7	0.164541189	6.036491238	0.17311501	7.59130577	1.004884579	1.078257373
Mystroxylon_20min	Experimental	Tree bark (*Mystroxylon* sp.)	20	8	0.16182948	6.15512861	0.168837925	8.696912365	1.004738403	1.007042047
Sediment_in_unused	Experimental	- -	0	1	0.109814485	8.829238905	1.120224916	12.81568736	1.0021637	0.539648064
Sediment_in_unused	Experimental	- -	0	2	0.101390314	9.753464808	0.668546058	13.40988397	1.003027982	0.748825493
Sediment_in_unused	Experimental	- -	0	3	0.139812902	8.471569997	0.640995327	13.2127618	1.003027982	0.748825493
Sediment_in_unused	Experimental	- -	0	4	0.123365066	41.55297117	0.729243268	13.61499002	1.003408042	0.827430603
Sediment_in_unused	Experimental	- -	0	5	0.143114904	8.640147759	0.619296908	11.15373414	1.004823645	1.21129648
Sediment_in_unused	Experimental	- -	0	6	0.113371616	7.071961039	0.339484173	12.63326226	1.007008835	1.73280502
Sediment_in_unused	Experimental	- -	0	7	0.123191207	3.090503757	0.462162412	11.72677569	1.007008835	1.73280502

Specimen ID	Context	Worked material	Time used (min)	Area_ID	Sq (µm)	Sal (µm)	Spc (1/µm)	Smr1 (%)	Y_max	Asfc
Sediment_in_unused	Experimental	- -	0	8	0.107681866	7.85683113	0.351817133	12.05244446	1.00399193	1.012514849
Sediment_in_10min	Experimental	Dry sediment inside BC	10	1	1.097293869	12.31999139	0.596274466	11.85160683	1.050164315	7.510872031
Sediment_in_10min	Experimental	Dry sediment inside BC	10	2	0.951610318	12.90881375	0.421405259	9.574858509	1.053030844	8.036461003
Sediment_in_10min	Experimental	Dry sediment inside BC	10	3	0.995619119	13.16421289	0.459392513	9.740776323	1.053030844	8.036461003
Sediment_in_10min	Experimental	Dry sediment inside BC	10	4	1.041260642	20.37968363	0.887464602	8.761669902	1.092890949	13.74083728
Sediment_in_10min	Experimental	Dry sediment inside BC	10	5	1.060828435	10.32204149	0.727463187	10.43512429	1.075225266	11.00117442
Sediment_in_10min	Experimental	Dry sediment inside BC	10	6	1.233029933	11.86474738	0.755528239	9.947861399	1.069732099	10.56503834
Sediment_in_10min	Experimental	Dry sediment inside BC	10	7	1.079643582	10.90281543	0.603959472	10.58713723	1.069732099	10.56503834
Sediment_in_10min	Experimental	Dry sediment inside BC	10	8	1.28202237	13.39700974	0.976221831	12.62802848	1.048122174	7.535809911
Sediment_in_20min	Experimental	Dry sediment inside BC	20	1	1.519791274	13.96425286	4.947525838	11.25685015	1.06565691	11.49408477
Sediment_in_20min	Experimental	Dry sediment inside BC	20	2	2.019973594	13.26588497	5.589300881	11.7107709	1.071366308	11.60826469
Sediment_in_20min	Experimental	Dry sediment inside BC	20	3	1.55427939	13.82162371	4.557276424	12.28917638	1.071366308	11.60826469
Sediment_in_20min	Experimental	Dry sediment inside BC	20	4	1.278096927	9.988996701	4.768279786	12.13242818	1.071245692	11.28441791
Sediment_in_20min	Experimental	Dry sediment inside BC	20	5	1.239491044	19.30018105	0.95623483	8.820253679	1.087148794	12.88170734
Sediment_in_20min	Experimental	Dry sediment inside BC	20	6	1.249319365	14.13843942	1.143715322	8.37306114	1.127341078	16.38056502
Sediment_in_20min	Experimental	Dry sediment inside BC	20	7	1.83944103	13.0490697	0.844791082	9.856941747	1.127341078	16.38056502
Sediment_in_20min	Experimental	Dry sediment inside BC	20	8	1.279492421	13.2451833	0.854921399	10.52249412	1.080184192	11.87391161
Sediment_out_unused	Experimental	- -	0	1	0.109275312	6.479843531	0.179173251	11.24386337	1.002121823	0.513058275
Sediment_out_unused	Experimental	- -	0	2	0.077708694	4.471478434	0.175115066	11.20911467	1.003560501	0.875318753
Sediment_out_unused	Experimental	- -	0	3	0.101630437	6.270137184	0.191753751	10.25490232	1.003560501	0.875318753
Sediment_out_unused	Experimental	- -	0	4	0.094767553	16.89715537	0.184118649	12.56158974	1.002889354	0.700888171
Sediment_out_unused	Experimental	- -	0	5	0.10382714	14.62056465	0.189394396	12.28264094	1.003594998	0.861587726
Sediment_out_unused	Experimental	- -	0	6	0.107220928	4.614272043	0.189359522	11.51797379	1.005622427	1.28774368
Sediment_out_unused	Experimental	- -	0	7	0.12985018	3.814344802	0.193931006	12.82983961	1.005622427	1.28774368
Sediment_out_unused	Experimental	- -	0	8	0.098967763	4.080479458	0.175691584	11.71314586	1.003890111	0.915846797
Sediment_out_10min	Experimental	Humic sediment outside BC	10	1	0.6273537	8.8342198	0.281971184	12.78141717	1.026410024	4.122779639
Sediment_out_10min	Experimental	Humic sediment outside BC	10	2	0.658020126	7.931429067	0.188976208	10.67514875	1.037672755	6.059940993
Sediment_out_10min	Experimental	Humic sediment outside BC	10	3	0.812489524	8.681129267	0.283462533	11.71328943	1.037672755	6.059940993
Sediment_out_10min	Experimental	Humic sediment outside BC	10	4	0.628444496	8.308155058	0.305181415	11.212487	1.028571889	4.919693351
Sediment_out_10min	Experimental	Humic sediment outside BC	10	5	0.828331196	10.77670596	0.416696639	9.874540251	1.061182216	9.725763109
Sediment_out_10min	Experimental	Humic sediment outside BC	10	6	1.015880772	11.69815159	0.499691556	9.521569812	1.027456665	4.66918619
Sediment_out_10min	Experimental	Humic sediment outside BC	10	7	0.623298674	9.092653534	0.372152224	8.311428367	1.027456665	4.66918619
Sediment_out_10min	Experimental	Humic sediment outside BC	10	8	0.816063725	31.5357378	0.412274838	9.465335245	1.036210114	5.460665249
Sediment_out_20min	Experimental	Humic sediment outside BC	20	1	0.711512498	9.385043424	0.604349087	10.11369104	1.044866782	7.752966966
Sediment_out_20min	Experimental	Humic sediment outside BC	20	2	0.763647968	9.038077149	0.536389877	9.353330969	1.05379671	9.304643349
Sediment_out_20min	Experimental	Humic sediment outside BC	20	3	0.939658366	12.32116743	0.717610705	12.09006872	1.05379671	9.304643349
Sediment_out_20min	Experimental	Humic sediment outside BC	20	4	1.130624424	11.62103991	0.749039538	12.49084087	1.067563311	11.04150606
Sediment_out_20min	Experimental	Humic sediment outside BC	20	5	1.072936073	11.48723584	1.328838733	10.28724292	1.059978486	9.380488264
Sediment_out_20min	Experimental	Humic sediment outside BC	20	6	1.095319467	11.53779338	0.95423676	12.70574185	1.080440819	12.19969664
Sediment_out_20min	Experimental	Humic sediment outside BC	20	7	1.112021618	9.923500963	0.849432587	11.03138937	1.080440819	12.19969664
Sediment_out_20min	Experimental	Humic sediment outside BC	20	8	1.092339768	12.00351888	1.215383722	11.42528767	1.073784263	11.78241937
Skin_unused	Experimental	- -	0	1	0.159340006	10.38071258	1.001515517	11.48558625	1.005023295	1.010121041
Skin_unused	Experimental	- -	0	2	0.170284097	11.23333112	0.898638534	12.335124	1.005901961	1.241808362
Skin_unused	Experimental	- -	0	3	0.12695763	6.02615583	1.081858229	9.689774494	1.006246711	1.240144733
Skin_unused	Experimental	- -	0	4	0.190358453	15.15167983	2.58046779	11.90786279	1.00495669	1.031224961
Skin_unused	Experimental	- -	0	5	0.11780157	7.0532163	0.674427326	11.22084106	1.00247451	0.479722882
Skin_unused	Experimental	- -	0	6	0.094757678	6.832124199	0.490913698	10.38302658	1.001861914	0.377470041
Skin_unused	Experimental	- -	0	7	0.115845082	7.482919683	0.612541667	11.21513776	1.001846766	0.352189652
Skin_unused	Experimental	- -	0	8	0.096313882	7.910708469	0.753325956	10.74302124	1.002347735	0.446374472
Skin_10min	Experimental	Rabbit skin	10	1	0.275563903	6.822805145	0.730171208	10.87618446	1.010730509	1.959620724
Skin_10min	Experimental	Rabbit skin	10	2	0.240040802	7.115046282	3.185041553	17.523576	1.008309864	1.550189414
Skin_10min	Experimental	Rabbit skin	10	3	0.266627713	7.754280456	0.828370153	10.04783699	1.006855466	1.264431235
Skin_10min	Experimental	Rabbit skin	10	4	0.200877121	6.788052816	0.726838158	12.98164119	1.010456062	1.933294768
Skin_10min	Experimental	Rabbit skin	10	5	0.136232569	5.272984886	0.385105656	10.83708328	1.00393949	0.795780146
Skin_10min	Experimental	Rabbit skin	10	6	0.133710742	5.94764024	0.282706541	11.9098669	1.003436794	0.67187596
Skin_10min	Experimental	Rabbit skin	10	7	0.159729764	5.634525025	0.362838546	11.38697028	1.00512755	0.994685839
Skin_10min	Experimental	Rabbit skin	10	8	0.170743239	4.87968218	0.6995073	10.75972566	1.004734987	0.912567738
Skin_20min	Experimental	Rabbit skin	20	1	0.224548115	7.273886309	1.19306602	9.747254778	1.00694277	1.286598041
Skin_20min	Experimental	Rabbit skin	20	2	0.21870786	6.049017682	0.904564352	10.44933769	1.006463017	1.181358958
Skin_20min	Experimental	Rabbit skin	20	3	0.274463457	6.597554605	1.024022103	9.296578622	1.007674161	1.43744454
Skin_20min	Experimental	Rabbit skin	20	4	0.226761947	5.725332242	0.674618209	10.37138581	1.009216707	1.680627677
Skin_20min	Experimental	Rabbit skin	20	5	0.359737687	8.559529435	0.655681144	14.61106472	1.012057737	2.038665462
Skin_20min	Experimental	Rabbit skin	20	6	0.193286631	6.433945356	1.087870913	10.23147361	1.006570619	1.297267822
Skin_20min	Experimental	Rabbit skin	20	7	0.300554896	8.746851264	0.982390151	10.78614535	1.005869572	1.236364125
Skin_20min	Experimental	Rabbit skin	20	8	0.175934812	6.482604188	0.764863027	10.3414238	1.009802501	1.73416245
Ochre_skin_unused	Experimental	- -	0	1	0.104950891	6.673737399	0.442655153	11.07595257	1.002471062	0.563269527
Ochre_skin_unused	Experimental	- -	0	2	0.106213228	7.239543463	0.618202121	11.87918348	1.003294213	0.80415358
Ochre_skin_unused	Experimental	- -	0	3	0.198810344	8.642647136	2.830028482	13.7898213	1.002697431	0.666477787
Ochre_skin_unused	Experimental	- -	0	4	0.095055873	8.366325234	0.525564472	12.81928809	1.006131518	0.935901138
Ochre_skin_unused	Experimental	- -	0	5	0.158261972	4.869199291	0.921893051	15.21844542	1.007573889	1.956602673
Ochre_skin_unused	Experimental	- -	0	6	0.11380474	6.03322673	0.75515186	11.8751362	1.007184912	1.982624693
Ochre_skin_unused	Experimental	- -	0	7	0.270695959	5.180229361	5.304819987	15.91859815	1.005486721	1.491896005
Ochre_skin_unused	Experimental	- -	0	8	0.106026813	6.375376743	0.791956476	12.39576716	1.010924335	1.759995995
Ochre_skin_10min	Experimental	Rabbit skin with ochre	10	1	0.294612966	7.917299056	0.332745287	8.263584978	1.005970251	0.868967494
Ochre_skin_10min	Experimental	Rabbit skin with ochre	10	2	0.363772315	9.72629731	0.553853142	9.344619956	1.007897288	1.160791586
Ochre_skin_10min	Experimental	Rabbit skin with ochre	10	3	0.350463996	9.256434107	0.856542621	9.473881623	1.008041918	1.299725034
Ochre_skin_10min	Experimental	Rabbit skin with ochre	10	4	0.310248749	8.320148498	0.665962542	9.040724091	1.009240572	1.636840775
Ochre_skin_10min	Experimental	Rabbit skin with ochre	10	5	0.340405077	10.42232938	0.906355259	8.46521676	1.007584331	1.162251115
Ochre_skin_10min	Experimental	Rabbit skin with ochre	10	6	0.432768336	13.06815443	0.688962088	10.25985084	1.009792158	1.410566739

Specimen ID	Context	Worked material	Time used (min)	Area_ID	Sq (µm)	Sal (µm)	Spc (1/µm)	Smr1 (%)	Y_max	Asfc
Ochre_skin_10min	Experimental	Rabbit skin with ochre	10	7	0.33912721	9.793604242	0.654160166	10.12483334	1.007344124	1.285956811
Ochre_skin_10min	Experimental	Rabbit skin with ochre	10	8	0.319893015	10.24104052	0.868707241	9.862252958	1.007313686	1.177380722
Ochre_skin_20min	Experimental	Rabbit skin with ochre	20	1	0.93200416	11.67169129	2.384643262	8.936656179	1.044236245	6.628208602
Ochre_skin_20min	Experimental	Rabbit skin with ochre	20	2	0.516274769	9.568483332	1.105332974	8.980202153	1.023660738	4.243934654
Ochre_skin_20min	Experimental	Rabbit skin with ochre	20	3	0.699258811	9.276439343	1.746420791	9.836926874	1.018182974	3.278832869
Ochre_skin_20min	Experimental	Rabbit skin with ochre	20	4	0.475731974	8.95099175	0.859303343	9.540590432	1.030449829	4.896092853
Ochre_skin_20min	Experimental	Rabbit skin with ochre	20	5	0.586858093	9.06629597	2.115611482	10.4132969	1.029391003	5.587906675
Ochre_skin_20min	Experimental	Rabbit skin with ochre	20	6	0.508506503	9.323285319	1.798639061	9.08767524	1.024498429	4.734167985
Ochre_skin_20min	Experimental	Rabbit skin with ochre	20	7	0.64183963	9.916569765	2.405779718	8.294152537	1.028042242	5.243618051
Ochre_skin_20min	Experimental	Rabbit skin with ochre	20	8	0.52798568	8.748276657	2.287169745	9.459171507	1.032976006	6.175999954
1943.13.3	Ethnographic	Tree bark (*Quercus* sp.)	- -	1	0.274551072	9.892896952	0.105219192	8.061618435	1.005798416	0.952470616
1943.13.3	Ethnographic	Tree bark (*Quercus* sp.)	- -	2	0.196409896	10.43343792	0.113822761	10.30136784	1.003688191	0.659657385
1943.13.3	Ethnographic	Tree bark (*Quercus* sp.)	- -	3	0.234102117	10.27361551	0.111649391	9.62533596	1.003780756	0.657355626
1943.13.3	Ethnographic	Tree bark (*Quercus* sp.)	- -	4	0.23267364	11.19479278	0.108857652	12.36861715	1.004713281	0.831963371
1943.13.3	Ethnographic	Tree bark (*Quercus* sp.)	- -	5	0.491566681	9.833438756	0.162221714	9.70530983	1.01474544	2.327862194
1943.13.3	Ethnographic	Tree bark (*Quercus* sp.)	- -	6	0.286332635	11.37075009	0.255945092	11.85706041	1.007346632	1.273823961
1943.13.3	Ethnographic	Tree bark (*Quercus* sp.)	- -	7	0.58549333	11.62767837	0.207569966	7.651497028	1.008005055	1.310690918
1943.13.3	Ethnographic	Tree bark (*Quercus* sp.)	- -	8	0.331843679	10.54970472	0.147637672	10.97994694	1.019424891	2.967842937
1943.13.3	Ethnographic	Tree bark (*Quercus* sp.)	- -	9	0.276985718	10.56625485	0.137234465	10.14131335	1.00659816	1.151739744
1943.13.3	Ethnographic	Tree bark (*Quercus* sp.)	- -	10	0.445729517	13.04788378	0.183296358	10.46845496	1.010911456	1.687976497
1943.13.3	Ethnographic	Tree bark (*Quercus* sp.)	- -	11	0.334367343	11.08020767	0.144822193	8.938262658	1.020519302	2.916136105
1943.13.3	Ethnographic	Tree bark (*Quercus* sp.)	- -	12	0.634741104	17.73363219	0.668332428	11.23942497	1.008233573	1.378993983
1943.13.3	Ethnographic	Tree bark (*Quercus* sp.)	- -	13	0.314766886	14.08707417	0.445282101	11.64332249	1.012413185	2.43631255
1943.13.3	Ethnographic	Tree bark (*Quercus* sp.)	- -	14	0.221854089	30.53962608	0.415624316	9.659386476	1.007542084	1.432703654
1943.13.3	Ethnographic	Tree bark (*Quercus* sp.)	- -	15	0.30080977	12.98218113	0.302260219	10.59080678	1.012559672	2.729336024
1943.13.3	Ethnographic	Tree bark (*Quercus* sp.)	- -	16	0.219595642	28.17410201	7.567905379	11.9659028	1.013025178	2.771458943
1943.13.3	Ethnographic	Tree bark (*Quercus* sp.)	- -	17	0.325727176	16.20433913	1.390057375	10.71696595	1.012639439	2.412081994
1943.13.3	Ethnographic	Tree bark (*Quercus* sp.)	- -	18	0.226742992	10.58604847	0.210806918	9.698333931	1.006164163	1.204719899
1943.13.3	Ethnographic	Tree bark (*Quercus* sp.)	- -	19	0.323420294	27.9400459	1.372103448	9.624002343	1.011234117	1.604651931
1943.13.3	Ethnographic	Tree bark (*Quercus* sp.)	- -	20	0.272432899	27.08080562	0.278497938	9.516332739	1.014136796	2.730730071
1940.30.58	Ethnographic	Tree bark (*Quercus* sp.)	- -	1	0.347493405	8.951764081	0.211624257	10.04430181	1.012977722	2.409971995
1940.30.58	Ethnographic	Tree bark (*Quercus* sp.)	- -	2	0.426150197	9.419734573	0.272864694	11.99439116	1.021035217	3.996973104
1940.30.58	Ethnographic	Tree bark (*Quercus* sp.)	- -	3	0.302216369	8.88544157	0.185278693	10.6560388	1.016909552	3.259934929
1940.30.58	Ethnographic	Tree bark (*Quercus* sp.)	- -	4	0.376224068	10.28200185	0.209214875	11.15472675	1.01057619	2.077801201
1940.30.58	Ethnographic	Tree bark (*Quercus* sp.)	- -	5	0.413465617	10.71622208	0.258305318	11.34488621	1.015987826	2.939856686
1940.30.58	Ethnographic	Tree bark (*Quercus* sp.)	- -	6	0.463708003	8.456258021	0.302828889	10.23160437	1.02234117	4.053796475
1940.30.58	Ethnographic	Tree bark (*Quercus* sp.)	- -	7	0.425017997	11.02142331	0.412878734	10.13338796	1.021698357	3.512641692
1940.30.58	Ethnographic	Tree bark (*Quercus* sp.)	- -	8	0.461322277	20.8576009	0.578159658	10.09621274	1.015548738	2.805434068
1940.30.58	Ethnographic	Tree bark (*Quercus* sp.)	- -	9	0.377804048	8.926890282	0.235366151	8.995087974	1.016405895	3.132317354
1940.30.58	Ethnographic	Tree bark (*Quercus* sp.)	- -	10	0.720007457	9.251559378	0.411318301	11.52012423	1.038268671	6.42013548
1940.30.58	Ethnographic	Tree bark (*Quercus* sp.)	- -	11	0.335074781	9.942347191	0.219851133	8.035479889	1.02063968	3.766573311
1940.30.58	Ethnographic	Tree bark (*Quercus* sp.)	- -	12	0.511621901	14.61800059	0.485601666	10.49367536	1.012689615	2.452995078
1940.30.58	Ethnographic	Tree bark (*Quercus* sp.)	- -	13	0.459023571	11.52104312	0.700333588	12.38269623	1.029655338	6.217785448
1940.30.58	Ethnographic	Tree bark (*Quercus* sp.)	- -	14	0.43626472	8.443064271	0.590800085	13.34729145	1.033259149	7.179623956
1940.30.58	Ethnographic	Tree bark (*Quercus* sp.)	- -	15	0.82874955	10.92878684	0.86561552	12.92642332	1.058860901	10.36123858
1940.30.58	Ethnographic	Tree bark (*Quercus* sp.)	- -	16	0.80341677	13.94973049	1.104596785	12.52007861	1.052033023	9.163002067
1940.30.58	Ethnographic	Tree bark (*Quercus* sp.)	- -	17	0.3987491	10.69094756	0.284515809	13.95838051	1.016885269	3.237481211
1940.30.58	Ethnographic	Tree bark (*Quercus* sp.)	- -	18	0.560549032	8.177177797	0.391068607	15.22440302	1.036826692	7.159797537
1940.30.58	Ethnographic	Tree bark (*Quercus* sp.)	- -	19	0.557174313	8.612867516	0.495604559	14.3767432	1.043969097	8.070974349
1940.30.58	Ethnographic	Tree bark (*Quercus* sp.)	- -	20	0.699768641	10.38272432	0.437378007	15.33760123	1.03532987	6.795852417
1940.30.56	Ethnographic	Tree bark (*Quercus* sp.)	- -	1	0.684801028	12.37600699	0.399075868	12.80263603	1.027592957	4.363981308
1940.30.56	Ethnographic	Tree bark (*Quercus* sp.)	- -	2	0.597943376	9.314313324	0.252423312	10.10645273	1.029057629	4.995928886
1940.30.56	Ethnographic	Tree bark (*Quercus* sp.)	- -	3	0.575157228	12.9751239	0.267902104	9.910234994	1.030825748	5.151703938
1940.30.56	Ethnographic	Tree bark (*Quercus* sp.)	- -	4	0.684192098	10.51799446	0.337722718	10.79634953	1.023716388	3.764941752
1940.30.56	Ethnographic	Tree bark (*Quercus* sp.)	- -	5	0.304016537	8.248392989	0.294990191	8.665397814	1.012233277	2.410877981
1940.30.56	Ethnographic	Tree bark (*Quercus* sp.)	- -	6	0.308608335	8.910711158	0.246703541	9.652932295	1.012515233	2.396671639
1940.30.56	Ethnographic	Tree bark (*Quercus* sp.)	- -	7	0.292664043	18.34383018	0.285087052	8.819826678	1.016024387	3.165059335
1940.30.56	Ethnographic	Tree bark (*Quercus* sp.)	- -	8	0.365130361	10.37390547	0.272115656	9.692084012	1.013472005	2.847292769
1940.30.56	Ethnographic	Tree bark (*Quercus* sp.)	- -	9	0.714887956	9.833156124	0.32475831	11.38967819	1.022604024	3.093498471
1940.30.56	Ethnographic	Tree bark (*Quercus* sp.)	- -	10	0.39904881	10.62064469	0.181448495	9.806879558	1.010721386	1.805633055
1940.30.56	Ethnographic	Tree bark (*Quercus* sp.)	- -	11	0.638451095	10.12938598	0.426965631	13.59033105	1.014655561	2.58464852
1940.30.56	Ethnographic	Tree bark (*Quercus* sp.)	- -	12	0.396582164	8.87204491	0.186443696	12.64727538	1.020718499	3.072199966
1940.30.56	Ethnographic	Tree bark (*Quercus* sp.)	- -	13	0.394703038	10.32747924	0.418903846	14.13141848	1.028765459	6.153555743
1940.30.56	Ethnographic	Tree bark (*Quercus* sp.)	- -	14	0.426852477	9.198935184	0.380378764	12.38474535	1.0305338	6.646842251
1940.30.56	Ethnographic	Tree bark (*Quercus* sp.)	- -	15	0.525393451	9.20818874	0.56181836	12.18581107	1.041575079	8.978643857
1940.30.56	Ethnographic	Tree bark (*Quercus* sp.)	- -	16	0.496005083	8.799759611	0.460434159	12.90169077	1.04281196	8.909694313
1940.30.56	Ethnographic	Tree bark (*Quercus* sp.)	- -	17	0.455131385	8.655846114	0.379625641	12.56709631	1.029554287	5.969373275
1940.30.56	Ethnographic	Tree bark (*Quercus* sp.)	- -	18	0.506597346	10.36615971	0.391897632	14.87171275	1.035977784	7.412376999
1940.30.56	Ethnographic	Tree bark (*Quercus* sp.)	- -	19	0.461958508	9.934576223	0.503265675	14.74753752	1.033162635	7.285607852
1940.30.56	Ethnographic	Tree bark (*Quercus* sp.)	- -	20	0.39987488	9.10860381	0.372424015	15.16820966	1.03273777	6.893109774
1940.30.56	Ethnographic	Tree bark (*Quercus* sp.)	- -	21	0.712773718	11.10592084	0.99430255	12.27535053	1.030377084	4.956983708
1940.30.56	Ethnographic	Tree bark (*Quercus* sp.)	- -	22	0.583559152	10.16074006	0.353997786	13.63837115	1.023860139	4.038686822
1940.30.56	Ethnographic	Tree bark (*Quercus* sp.)	- -	23	0.615385693	11.63063276	0.616981467	11.58802753	1.035872262	6.094346909
1940.30.56	Ethnographic	Tree bark (*Quercus* sp.)	- -	24	0.721187153	10.62108641	0.741484037	13.10300953	1.027098121	4.77748338
1940.30.56	Ethnographic	Tree bark (*Quercus* sp.)	- -	25	0.41328596	9.453582018	0.272983261	8.995522553	1.015166768	2.720471607
1940.30.56	Ethnographic	Tree bark (*Quercus* sp.)	- -	26	0.437126314	9.720425152	0.281959089	11.62096163	1.016695951	2.910311702
1940.30.56	Ethnographic	Tree bark (*Quercus* sp.)	- -	27	0.430150958	9.215909352	0.397124113	11.07903976	1.012396341	2.192458598
1940.30.56	Ethnographic	Tree bark (*Quercus* sp.)	- -	28	0.376675614	9.529128959	0.222661673	11.05505517	1.014668841	2.459989023

Specimen ID	Context	Worked material	Time used (min)	Area_ID	Sq (µm)	Sal (µm)	Spc (1/µm)	Smr1 (%)	Y_max	Asfc
1940.30.56	Ethnographic	Tree bark (*Quercus* sp.)	- -	29	0.745020281	10.32911556	0.598077814	17.72709998	1.041299689	7.123026771
1940.30.56	Ethnographic	Tree bark (*Quercus* sp.)	- -	30	0.642388768	7.431683218	0.435068764	17.62511264	1.040693375	7.884939096
1940.30.56	Ethnographic	Tree bark (*Quercus* sp.)	- -	31	0.389067698	8.395935153	0.32605867	13.15709137	1.028828432	5.512211823
1940.30.56	Ethnographic	Tree bark (*Quercus* sp.)	- -	32	0.536147198	12.19952727	0.368244166	14.9014185	1.022690187	4.605262868
1940.30.54	Ethnographic	Tree bark (*Quercus* sp.)	- -	1	0.496728667	15.75977578	0.484057814	10.79474688	1.016394975	2.390028836
1940.30.54	Ethnographic	Tree bark (*Quercus* sp.)	- -	2	0.538220776	17.5979723	1.179270697	10.78909415	1.015921799	2.233389309
1940.30.54	Ethnographic	Tree bark (*Quercus* sp.)	- -	3	0.480359386	12.53830213	0.525872094	9.15036768	1.010526386	1.830693343
1940.30.54	Ethnographic	Tree bark (*Quercus* sp.)	- -	4	0.343738192	13.90427514	0.265501	8.982243783	1.014642522	2.334448201
1940.30.54	Ethnographic	Tree bark (*Quercus* sp.)	- -	5	0.600109987	10.57730555	0.943382597	13.80636937	1.020625916	3.020639455
1940.30.54	Ethnographic	Tree bark (*Quercus* sp.)	- -	6	0.6670085	11.59602879	1.23172995	10.22249558	1.023976793	3.586537102
1940.30.54	Ethnographic	Tree bark (*Quercus* sp.)	- -	7	0.558265978	12.65752039	0.636298612	12.22976183	1.039678709	6.184646288
1940.30.54	Ethnographic	Tree bark (*Quercus* sp.)	- -	8	0.897502488	10.02117305	3.305751735	11.5980278	1.016571612	2.462834334
1940.30.54	Ethnographic	Tree bark (*Quercus* sp.)	- -	9	0.513631222	16.59156702	0.4664542	11.09485575	1.020420641	3.497996943
1940.30.54	Ethnographic	Tree bark (*Quercus* sp.)	- -	10	0.507496172	14.27617732	0.393005962	9.871673933	1.020336795	3.592574069
1940.30.54	Ethnographic	Tree bark (*Quercus* sp.)	- -	11	0.55708284	14.04271996	0.577576549	10.563666	1.018174891	2.950350018
1940.30.54	Ethnographic	Tree bark (*Quercus* sp.)	- -	12	0.463819024	27.84193332	0.874248683	9.172919496	1.019492062	3.199543836
1940.30.54	Ethnographic	Tree bark (*Quercus* sp.)	- -	13	0.428341874	12.45344636	0.29524715	11.5933902	1.013694874	2.333151197
1940.30.54	Ethnographic	Tree bark (*Quercus* sp.)	- -	14	0.470447918	14.88705065	0.453042816	13.08981179	1.015498351	2.501177545
1940.30.54	Ethnographic	Tree bark (*Quercus* sp.)	- -	15	0.825706814	12.18182447	1.219947849	11.54664792	1.057160701	7.704303617
1940.30.54	Ethnographic	Tree bark (*Quercus* sp.)	- -	16	1.045355341	13.36328306	1.548867275	14.19624289	1.030963579	4.535356294
1940.30.54	Ethnographic	Tree bark (*Quercus* sp.)	- -	17	0.684801028	23.30420804	0.925830098	11.2181104	1.020260379	3.480149919
1940.30.54	Ethnographic	Tree bark (*Quercus* sp.)	- -	18	0.597943376	53.98806664	2.213934695	9.605243895	1.029803286	3.199469378
1940.30.54	Ethnographic	Tree bark (*Quercus* sp.)	- -	19	0.575157228	11.16725572	0.782431417	8.882962691	1.032669402	5.626691672
1940.30.54	Ethnographic	Tree bark (*Quercus* sp.)	- -	20	0.684192098	15.44085144	1.329126648	11.36138126	1.021680133	3.778972714
B5b_RGS	Archaeological	- -	- -	1	3.440133429	14.44969019	2.49383558	10.70284222	1.300207715	39.19111908
B5b_RGS	Archaeological	- -	- -	2	2.743520164	11.97196038	1.567585266	10.39261705	1.373845097	38.79014373
B5b_RGS	Archaeological	- -	- -	3	3.563396539	13.37384334	2.115983669	11.04422981	1.373845097	38.79014373
B5b_RGS	Archaeological	- -	- -	4	3.18921277	12.44573083	1.859553254	12.65573133	1.354099035	41.20956186
B5b_RGS	Archaeological	- -	- -	5	2.511297631	11.01372977	1.564252122	11.34523679	1.292486923	38.39885717
B5b_RGS	Archaeological	- -	- -	6	2.885194581	11.47298938	1.694659693	12.3885268	1.303165984	40.77390563
B5b_RGS	Archaeological	- -	- -	7	2.61009878	13.8656668	1.630102069	10.1446198	1.303165984	40.77390563
B5b_RGS	Archaeological	- -	- -	8	3.101019562	10.98009664	2.040476479	12.78281913	1.328352877	43.21376432
B4d_BS6	Archaeological	- -	- -	1	1.24577481	22.86641689	0.843716381	10.45675716	1.078845979	12.98752404
B4d_BS6	Archaeological	- -	- -	2	1.075470688	17.09779675	0.814095	10.82410564	1.099231092	16.26258214
B4d_BS6	Archaeological	- -	- -	3	1.217374537	16.02329609	0.888173955	10.57907639	1.099231092	16.26258214
B4d_BS6	Archaeological	- -	- -	4	1.137540837	11.97049427	0.820597931	11.11338768	1.081355597	13.02001694
B4d_BS6	Archaeological	- -	- -	5	1.996612145	11.67082008	1.575576636	12.04255771	1.251910234	37.89869801
B4d_BS6	Archaeological	- -	- -	6	2.036938158	11.7939784	1.370623406	12.27553694	1.306578882	41.28984319
B4d_BS6	Archaeological	- -	- -	7	2.675110945	11.89168133	1.507577844	12.60673795	1.306578882	41.28984319
B4d_BS6	Archaeological	- -	- -	8	2.757513825	13.0155349	1.744503427	16.17655958	1.284994569	35.9784224
C5d_BS12	Archaeological	- -	- -	1	1.155329487	12.7299009	1.031090162	12.66344451	1.104057164	15.47713557
C5d_BS12	Archaeological	- -	- -	2	1.243343233	12.56884523	1.024820848	11.87785759	1.105159096	12.56097464
C5d_BS12	Archaeological	- -	- -	3	1.354740574	19.40748111	1.299723588	11.29992201	1.105159096	12.56097464
C5d_BS12	Archaeological	- -	- -	4	1.497045758	27.06016577	1.412989197	14.2415295	1.10974575	13.84032635
C5d_BS12	Archaeological	- -	- -	5	1.394188426	15.30243916	1.196810863	11.92558454	1.112227012	15.18070846
C5d_BS12	Archaeological	- -	- -	6	1.29950342	13.14319762	1.547106974	12.01934016	1.41171787	32.93157367
C5d_BS12	Archaeological	- -	- -	7	4.877631129	11.99298117	2.439803711	19.33123624	1.41171787	32.93157367
C5d_BS12	Archaeological	- -	- -	8	1.600091189	12.32490334	1.324467946	11.92960738	1.09100684	9.591255657
B4a_PGS3	Archaeological	- -	- -	1	0.452356765	10.34768297	0.232421105	11.15905134	1.01105838	1.945571346
B4a_PGS3	Archaeological	- -	- -	2	0.389996151	10.46218744	0.197224003	10.722048	1.022677701	4.393430357
B4a_PGS3	Archaeological	- -	- -	3	0.45795773	11.32448476	0.374208588	9.724463476	1.022677701	4.393430357
B4a_PGS3	Archaeological	- -	- -	4	0.447855741	10.95269521	0.329255301	10.87097655	1.019576496	3.754315525
B4a_PGS3	Archaeological	- -	- -	5	0.501365271	13.73512416	0.585648083	9.958723845	1.024923156	3.568241494
B4a_PGS3	Archaeological	- -	- -	6	0.729809108	38.62284819	0.588269331	12.37766683	1.028788612	4.945499278
B4a_PGS3	Archaeological	- -	- -	7	0.621687776	13.15021251	0.490565523	11.57510582	1.028788612	4.945499278
B4a_PGS3	Archaeological	- -	- -	8	0.713091289	10.68904252	0.518852609	12.31621217	1.034566269	5.913027668
B4a_BS14	Archaeological	- -	- -	1	0.564667405	11.70848715	0.522139865	11.31028675	1.030983471	5.66077137
B4a_BS14	Archaeological	- -	- -	2	0.585486816	10.9651887	0.627530162	14.59136293	1.06279974	10.82277219
B4a_BS14	Archaeological	- -	- -	3	0.905745068	10.39610935	0.660214688	16.16610677	1.06279974	10.82277219
B4a_BS14	Archaeological	- -	- -	4	0.887791986	13.58935756	0.627920573	13.22880953	1.054172299	8.907291727
B4a_BS14	Archaeological	- -	- -	5	0.618049099	10.14403495	0.505845039	11.46516022	1.038493455	7.407582214
B4a_BS14	Archaeological	- -	- -	6	0.684264348	10.31888594	0.661453173	11.29358234	1.032314009	6.426024262
B4a_BS14	Archaeological	- -	- -	7	0.524892097	9.151321304	0.505354575	10.72668724	1.032314009	6.426024262
B4a_BS14	Archaeological	- -	- -	8	0.578439584	11.69456246	0.604607975	10.23053224	1.031498508	5.981617334
C5d_RGS	Archaeological	- -	- -	1	0.803059767	10.68861214	0.807298589	14.9283017	1.053969579	9.885497418
C5d_RGS	Archaeological	- -	- -	2	0.713929484	8.75792534	0.733135857	12.77452134	1.021167704	3.988710634
C5d_RGS	Archaeological	- -	- -	3	0.444211112	10.05292807	0.347436453	10.37231016	1.021167704	3.988710634
C5d_RGS	Archaeological	- -	- -	4	0.510540548	8.720637831	0.354327302	11.40247977	1.02803447	5.660129689
C5d_RGS	Archaeological	- -	- -	5	0.865651924	8.164522049	0.476280398	13.96749142	1.033473263	5.400247597
C5d_RGS	Archaeological	- -	- -	6	0.589061289	11.34980826	0.400142381	15.12159622	1.058013531	7.774738279
C5d_RGS	Archaeological	- -	- -	7	0.985879337	15.9998556	0.736772208	13.15158264	1.058013531	7.774738279
C5d_RGS	Archaeological	- -	- -	8	0.697937422	16.91210799	0.473960069	13.09686822	1.030171341	4.529945783
B4b_LBG	Archaeological	- -	- -	1	0.984053004	9.720484268	0.610124185	13.73643412	1.039269574	6.791100972
B4b_LBG	Archaeological	- -	- -	2	0.685223413	11.97169518	0.575384466	12.12987525	1.058187891	10.42600519
B4b_LBG	Archaeological	- -	- -	3	0.775535656	11.64729343	0.526017495	13.37686909	1.058187891	10.42600519
B4b_LBG	Archaeological	- -	- -	4	0.731381971	21.20128738	0.671655773	12.81788182	1.049030982	7.882604078
B4b_LBG	Archaeological	- -	- -	5	0.735662044	12.28269018	0.473519985	10.55448556	1.038346042	6.498426069

Specimen ID	Context	Worked material	Time used (min)	Area_ID	Sq (µm)	Sal (µm)	Spc (1/µm)	Smr1 (%)	Y_max	Asfc
B4b_LBG	Archaeological	- -	- -	6	0.763295571	11.90073285	0.336032839	11.1668486	1.034662819	6.075144441
B4b_LBG	Archaeological	- -	- -	7	0.641499203	12.75174414	0.452520067	11.80013696	1.034662819	6.075144441
B4b_LBG	Archaeological	- -	- -	8	0.865343657	13.18926342	0.484438054	12.76307173	1.05185003	8.804323378
B5b_GR2	Archaeological	- -	- -	1	0.862982662	14.93498702	0.725995479	13.19070567	1.033516987	5.195668705
B5b_GR2	Archaeological	- -	- -	2	0.822904882	14.73049498	0.733899033	12.59018039	1.03166874	5.785687714
B5b_GR2	Archaeological	- -	- -	3	0.609902965	15.7208245	0.800396551	10.84532742	1.03166874	5.785687714
B5b_GR2	Archaeological	- -	- -	4	0.854849517	23.52179879	0.915350362	10.39918774	1.052637877	8.473679367
B5b_GR2	Archaeological	- -	- -	5	0.901366732	11.05256608	0.632950148	13.38169598	1.03035614	4.7251858
B5b_GR2	Archaeological	- -	- -	6	0.776677284	11.08898233	0.526881646	11.70415206	1.040484865	5.781210213
B5b_GR2	Archaeological	- -	- -	7	1.4832092	8.299232563	0.473433304	12.55047738	1.040484865	5.781210213
B5b_GR2	Archaeological	- -	- -	8	0.514233141	36.01901005	0.467303697	11.12221317	1.020320149	3.172308769
C4d_PGS2	Archaeological	- -	- -	1	0.569642394	10.37618807	0.797956649	12.26574864	1.04681514	7.905974655
C4d_PGS2	Archaeological	- -	- -	2	0.823694529	8.702131857	0.56149786	14.77667921	1.051918118	8.395142357
C4d_PGS2	Archaeological	- -	- -	3	0.969828837	7.730679636	0.881988669	13.24858086	1.051918118	8.395142357
C4d_PGS2	Archaeological	- -	- -	4	0.978967842	8.159264983	0.953463372	16.16697342	1.049247452	7.741746101
C4d_PGS2	Archaeological	- -	- -	5	1.070516466	19.4066371	0.902992833	12.16825966	1.063882358	10.32899938
C4d_PGS2	Archaeological	- -	- -	6	1.014343879	11.9611236	0.9355029	15.75672213	1.035925948	5.807009645
C4d_PGS2	Archaeological	- -	- -	7	0.787378057	10.87127229	1.127738623	11.67606163	1.035925948	5.807009645
C4d_PGS2	Archaeological	- -	- -	8	0.477179748	13.77968129	1.137003907	11.49702828	1.033840801	7.431372474
C4c_BS9	Archaeological	- -	- -	1	0.912324184	9.657524654	0.571139102	13.9364471	1.056102447	9.312270767
C4c_BS9	Archaeological	- -	- -	2	0.857836896	13.35558563	0.518483888	15.25628234	1.063192899	10.32254635
C4c_BS9	Archaeological	- -	- -	3	1.056555756	11.42910619	0.585270583	15.57310572	1.063192899	10.32254635
C4c_BS9	Archaeological	- -	- -	4	1.114345083	14.60571313	0.654529623	15.71665217	1.072550593	10.8200415
C4c_BS9	Archaeological	- -	- -	5	1.148185857	10.6994739	1.003410359	17.28137533	1.064616042	10.95799831
C4c_BS9	Archaeological	- -	- -	6	0.965871667	10.7544853	0.910377872	14.8634754	1.055036009	10.95018766
C4c_BS9	Archaeological	- -	- -	7	0.63573089	10.49421788	1.155563284	14.8317629	1.055036009	10.95018766
C4c_BS9	Archaeological	- -	- -	8	0.780068906	8.105942011	1.114887943	15.21352268	1.068508244	12.72615858
